# Exploring the Formation Kinetics of Octacalcium Phosphate from Alpha-Tricalcium Phosphate: Synthesis Scale-Up, Determination of Transient Phases, Their Morphology and Biocompatibility

**DOI:** 10.3390/biom13030462

**Published:** 2023-03-02

**Authors:** Ilijana Kovrlija, Ksenia Menshikh, Olivier Marsan, Christian Rey, Christèle Combes, Janis Locs, Dagnija Loca

**Affiliations:** 1 Rudolfs Cimdins Riga Biomaterials Innovation and Development Centre, Faculty of Materials Science and Applied Chemistry, Institute of General Chemical Engineering, Riga Technical University, Pulka 3, LV-1007 Riga, Latvia; 2 Center for Translational Research on Autoimmune and Allergic Disease—CAAD, Department of Health Sciences, Università del Piemonte Orientale, 28100 Novara, Italy; 3 CIRIMAT, Université de Toulouse, CNRS, Toulouse INP—ENSIACET, 4 Allée Emile Monso, CEDEX 4, 31030 Toulouse, France; 4 Baltic Biomaterials Centre of Excellence, Headquarters at Riga Technical University, LV-1007 Riga, Latvia

**Keywords:** octacalcium phosphate, α-TCP, DCPD, calcium phosphate, hydrolysis, scale-up, mesenchymal stem cells, FTIR, XRD, Raman

## Abstract

Even with decades of research studies behind octacalcium phosphate (OCP), determination of OCP phase formation has proved to be a cumbersome challenge. Even though obtaining a large quantity of OCP is important for potential clinical uses, it still remains a hindrance to obtain high yields of pure OCP. Taking that into consideration, the purpose of this study was to scale-up OCP synthesis for the first time and to use a multi-technique approach to follow the phase transformation pathway at multiple time points. In the present study, OCP has been synthesized from α-tricalcium phosphate (α-TCP), and subsequently scaled-up tenfold and hundredfold (100 mg → 10 g). The hydrolysis mechanism has been followed and described by using XRD and FTIR spectroscopy, as well as Raman and SEM. Gradual transformation into the OCP phase transpired through dicalcium phosphate dihydrate (brushite, DCPD, up to ~36%) as an intermediary phase. Furthermore, the obtained transitional phases and final OCP phases (across all scale-up levels) were tested with human bone marrow-derived mesenchymal stem cells (hBMSCs), in order to see how different phase mixtures affect the cell viability, and also to corroborate the safety of the scaled-up product. Twelve out of seventeen specimens showed satisfactory percentages of cell viability and confirmed the prospective use of scaled-up OCP in further in vitro studies. The present study, therefore, provides the first scale-up process of OCP synthesis, an in depth understanding of the formation pathway, and investigation of the parameters able to contribute in the OCP phase formation.

## 1. Introduction

Ever since calcium phosphate (CaP) started to gain scientific interest in 1770 [1], a revolution has taken place across several fields, ranging from chemistry and material science to biomedical applications. Many synthetic bone substitutes have been developed, but the limitation of the natural material, with its complex and non-stoichiometric calcium phosphate apatite structure, means they are still far out of reach from achieving the optimum goal. The main reasons being that the natural material contains many substitutes for calcium and phosphate ions and a great number of heterogeneities corresponding to the type of bone can influence it (trabecular or compact [2], biological diversity, remodelling rate, living style, etc [3,4,5,6]). However, the formation of non-stoichiometric apatites has emerged as a potential solution. One of the calcium phosphates that has been pushing itself to the forefront of material science and has also been considered as a bone mineral precursor [7,8,9] is octacalcium phosphate (Ca_8_(HPO_4_)_2_(PO_4_)_4_·5H_2_O; OCP). Its similarity to apatite stems from the characteristic apatite layers parallel to the (100) plane, with interlaying hydrated layers that are comprised of an alterable number of water molecules, which are found between more spatially spread out phosphate and calcium ions [10]. In addition, OCP can explain the existence of non-stoichiometry in apatites [3] through its ability to interlay with apatite domains [3]. Moreover, OCP has a plethora of other benefits such as solubility in close equilibrium with blood serum [11,12], substantial affinity towards organic molecules (helpful in biological mineralization, drug delivery platforms) [13], the tendency to convert to the thermodynamically more stable apatite [14], and the potential to promote osteoblastic cell differentiation [15]. 

Several improvements of OCP with different ions and drugs have been published [16,17]. There has also been evidence that an ultrahigh drug loading capacity can be accomplished if the drug loading is implemented in situ during the primary stages of CaP synthesis [18]. Conversely, if OCP is to be used as a drug delivery vehicle, the complexity of the formation pathway needs to be identified and substantiated. For that reason, significant efforts have been taken to develop the OCP synthesis processes. The precipitation route has been heavily employed and examined in more detail than the older, original hydrolysis route [19,20]. However, synthesis via precipitation has its own limitations, and with the already complicated chemistry of CaP formation, it can bring undesired outcomes. Several aspects ranging from the preparation of precursor solutions (e.g., calcium sources), incomplete reactions involving metastable phases, and even the impact of incomplete washing can have a negative effect on the precipitated product [21]. The excessive number of parameters (dose rate, stirring, purity of precursor salts) can trigger an already highly sensitive mechanism and cause an even greater inconvenience in establishing the phase purity. If the utilization of the CaP is being aimed at creating a drug delivery system, one must be careful of the synthesis environment due to the sensitivity of the chosen drug. All of the aforementioned reasons have pushed the decision to select the hydrolysis pathway of obtaining OCP as a preferential method [22,23,24]. Even though hydrolysis is a complex process [25], involving not only a precipitation control, but also a dissolution control, the existence of only one precursor could make the repeatability of the synthesis with different incorporated drugs more secure. As examples of precursors, α-tricalcium phosphate (α–TCP) and dicalcium phosphate dihydrate (CaHPO_4_·2H_2_O, DCPD or brushite) have been used in the literature [24,26,27,28]. The first experiments with α–TCP have been performed by Monma et al. [28], where they have tested different approaches to optimize the new (at that time) and simple innovative method of obtaining OCP. To our knowledge Monma, Graham, and Bigi were the only ones to study the effects of different synthesis parameters on the α–TCP transformation kinetic in more detail [24,28,29]. Utilization of a precursor such as α–TCP limits the pool of unwanted external ion incorporation (e.g., chloride ion (Cl^−^)), and if chosen, it could be performed at room temperature, which would also enable the easy in situ incorporation of ions and drugs [30,31]. However, the study of Graham and Brown was performed a while ago and the reaction process was determined only via the X-ray diffraction technique (XRD) and not by using a multiple technique approach to further check the composition of the formed OCP. Other obstacles that have been noticed throughout the literature review is the relatively small amount of the obtained product or no information on how much was obtained, as well as whether the synthesis yield is uniform across the performed characterization techniques. 

Having in mind the present limits, the main goal of the manuscript is to shed light on the chemical processes occurring during the α-TCP hydrolysis route towards obtaining OCP as a final product. The subsequent step that we tried to embark on was to perform a tenfold and a hundredfold scale-up of the reaction. Herein, we present a series of experiments displaying the kinetic transformation and a potential pathway of OCP formation from α-TCP as the initial raw material. Encouraged by the satisfactory preliminary agreement in all the experiments and throughout all the scale-up platforms, we have confirmed the results with a set of preselected characterization methods able to deliver the data on different levels (XRD, Fourier transform infrared (FTIR), Raman spectroscopy, and scanning electron microscopy (SEM)). As no research team has been able to deliver the quantifiable data of the OCP phase purity due to the strong resemblance with apatite, and as many of them do not present an entire picture, in the present manuscript we have assembled the data from the aforementioned techniques and created one joint thread among them. Determining the quantities of present phases with different techniques is still a big challenge, but providing a semi-quantitative approach with combining all the complementary methods is to be of significant value for the scientific community. Additionally, as the end goal of each biomaterial (used for bone tissue engineering) is to enhance the bone reconstruction/neoformation [32], attained final OCPs and the mix of phases from observed kinetic time points have undergone cytocompatibility tests with human bone marrow-derived mesenchymal stem cells (hBMSCs).

Within the current manuscript we have, for the first time, successfully performed a scale-up of the OCP synthesis process and proven the biocompatibility of the hundredfold scaled-up intermediate and final products. Furthermore, as OCP has great potential as a drug delivery vehicle, and as its mechanism of the formation pathway is of utmost importance for the in situ drug loading, we have provided a comprehensive physico-chemical overview of the phase interchange that transpire in the four described steps of the hydrolysis process of α-TCP to OCP. The data have been backed up with XRD, FTIR and Raman spectroscopy, BET, laser granulometry, and SEM in order to create a common thread of information that is usually found in scarce detail.

## 2. Materials and Methods

### 2.1. Materials

The following reagents have been used throughout the experiments: diammonium hydrogen phosphate ((NH_4_)_2_HPO_4_, ACS, M = 132.05 g/mol, Lot A1015407 628), calcium nitrate tetrahydrate (Ca(NO_3_)_2_·4H_2_O, ACS, M = 236.15 g/mol, Lot AM1339021 840), and sodium hydroxide (NaOH, ISO, M = 40.00 g/mol, Lot B1289298 624) were purchased from Emsure^®^ (Darmstadt, Germany). Orthophosphoric acid (H_3_PO_4_, 75%) was purchased from Latvijas Ķīmija, Riga, Latvia. Potassium bromide (KBr for IR spectroscopy, Uvasol^®^) was purchased from Merck KGaA, Darmstadt, Germany. Low-glucose as well as high-glucose Dulbecco’s modified Eagle’s medium (DMEM), fetal bovine serum (FBS), and Trypsin EDTA 1X were purchased from Sigma-Aldrich (St. Louis, MO, USA) while penicillin–streptomycin was purchased from Invitrogen (Waltham, MA, USA). Ethanol (EtOH, BioUltra, ≥99.8%, molecular biology grade, CAS: 64-17-5) was purchased from Sigma-Aldrich, USA. The 24-well plates (Ref. N. 30024) were purchased from SPL Life Sciences (Pochon, Republic of Korea). Resazurin sodium salt (Ref. N. R7017-5G, CAS: 62758-13-8) was purchased from Sigma-Aldrich, USA. 4′,6-Diamidino-2-phenylindole dihydrochloride (DAPI, Ref. N. 90229, CAS: 28718-90-3) was purchased from Millipore (St. Louis, MO, USA) and Alexa Flour 594-conjugated phalloidin (Ref. N. A12381) was purchased from Invitrogen. Tween^®^-20 detergent (Molecular biology grade, CAS: 9005-64-5) and paraformaldehyde (Reagent grade, crystalline, Ref. N. P6148-1KG, CAS: 30525-89-4) were purchased from Sigma-Aldrich, USA. Phosphate buffered saline (PBS) (N.P4417, Lot SLCH0989) was purchased from Sigma-Aldrich, USA. The cell line used for the research has the following specifications: human bone marrow-derived stem cells (hBMSCs, hTERT-BMSC clone Y201bMSCs), isolated previously by James et al. [33] from bone marrow and immortalized through hTERT lentiviral vectors.

### 2.2. Synthesis Methodology

#### 2.2.1. Synthesis of Amorphous Calcium Phosphate

To prepare α–TCP, later used for OCP formation, amorphous calcium phosphate (ACP) was heat treated. Hence, the synthesis of ACP is first presented. The wet precipitation method was used to synthesize the ACP powder with a Ca/P molar ratio of 1.5. Both 0.29 M (NH_4_)_2_HPO_4_ and 0.43 M Ca(NO_3_)_2_·4H_2_O were used for this purpose. The pH of prepared solutions was adjusted using 7 M NaOH. The solutions were poured into the beaker at the same time at room temperature and stirred with a magnetic stirrer. Upon the end of the synthesis, the suspension had a pH value of 9.1–9.4. To obtain the product, the suspension was filtered, rinsed, and frozen in liquid nitrogen. Prepared samples were lyophilized (BETA 2-8 LSCplus, Martin Christ Freeze Dryers, Osterode, Germany) for 72 h (1 mbar for primary drying stage and 0.0010 mbar for secondary drying stage).

#### 2.2.2. α-Tricalcium Phosphate Synthesis and Hydrolysis to Octacalcium Phosphate

To obtain α-TCP, the ACP was heat treated at 650 °C (heating rate 5 °C/min, holding time one hour) [34,35]. Prior to continuing with OCP synthesis, α-TCP was fully analyzed and its phase composition was confirmed with XRD. A total of 100 mg of α-TCP were immersed into 50 mL of 0.0016 M H_3_PO_4_ solution at room temperature, under unremitting stirring (300 rpm), during the 24 h period. pH was monitored throughout the entire time frame. After each selected time point (1 h, 3 h, 6 h, 10 h, 12 h, and 24 h), the collected suspension was centrifuged at 3000 rpm for 2 min, washed several times with deionized water, and dried overnight at 37 °C (Figure S1 in Supplementary Material (SM)). The same α-TCP used for the synthesis was operated as a reference for α-TCP in all measurements. The above described synthesis (100 mg) is further referred as the initial synthesis (IS). The syntheses have been performed more than five times in order to claim the repetitive formation pattern.

#### 2.2.3. Tenfold and Hundredfold Scale-Up

An identical preparation procedure of α-TCP powder was performed for both scale-up levels. The stirring rate for each level of synthesis was increased due to the increased volume. Based on the preliminary findings, after the preparatory tests, the time points for observation have been chosen. An amount of 1.0 g of α-TCP raw material was immersed into 500 mL of 0.0016 M H_3_PO_4_ (tenfold scale-up, referred to further on as the 10 × SC; 1 g yield). The suspension was under continuous stirring (400 rpm), during the 72-h period. Due to the geometric and volume limitations of the reactor, a used amount of α-TCP powder for the hundredfold scale-up (referred to further on as the 100 × SC; 10 g amount) was recalculated to the total volume of 4.5 L. Stirring was supplied through the overhead mixer (500 rpm) during the course of 180 h. For both set ups, pH was monitored throughout the entire duration of the synthesis. After each selected time point, the collected suspensions were centrifuged at 3000 rpm, for 2 min, washed with deionized water, and dried at 37 °C. The syntheses have been performed more than five times in order to claim the repetitive formation pattern. 

#### 2.2.4. Octacalcium Phosphate Formation Pathway

The kinetic transformation and mechanism of OCP formation were followed on each level (IS, 10 × SC and 100 × SC). For IS, samples have been taken at 1 h, 3 h, 6 h, 10 h, 12 h, and 24 h. For 10 × SC, samples have been taken at 24 h, 48 h, and 72 h. For 100 × SC, samples have been taken at multiple time points out of which 1 h, 24 h, 30 h, 48 h, 78 h, 96 h, 144 h, and 180 h have been focused on in the main manuscript, as they exhibited the important changes. The list of all analyzed samples at different time points and their presentation within the manuscript and SM have been described in Table S1. Because of the small initial amount of the α-TCP powder used, IS was performed individually for each time point. The amount of 50 mL of samples from tenfold and hundred-fold scale-ups was collected from a 600 mL and five L reactor, respectively. Each sample taken was operated according to the steps described in Section 2.2.2 and Section 2.2.3. Figure S1 in Supplementary Material summarizes the experiment methodology. 

### 2.3. Phase and Composition Characterization

#### 2.3.1. X-ray Diffraction 

The presence of crystalline phases in all obtained samples was examined by using X-ray powder diffractometry (XRD). XRD was performed using PANalytical Aeris diffractometer (The Netherlands) and accompanying analyses were performed with suitable software (X’Pert Data Collector, X’Pert Data Viewer, X’PertHighScore and the International Centre for Diffraction Data PDF-2 (ICDD) database). To obtain the XRD pattern, the following parameters were used: 40 kV and 15 mA, step size 0.0435°, within 2θ range from 3° to 60°, time per step 299.575 s. The quantitative amount of the observed phases was determined with the Rietveld refinement procedure by using Profex software [36]. For crystalline phase identification following, ICDD entries were used—#026-1056 for OCP, #01-072-1243 for hydroxyapatite (HAp), #009-0077 for DCPD, and #009-0348 for α-TCP. The analysis was also conducted on the performed preliminary synthesis in order to claim the repetitive formation pattern, and the final one was presented in the manuscript.

#### 2.3.2. Fourier-Transform Infrared Spectroscopy

The Fourier-transform infrared spectrometer Nicolet iS 50 (Thermo Scientific, Waltham, MA, USA) was used in transmission mode with the potassium bromide (KBr) pellet method, which was employed to characterize functional groups of powders at the molecular level. The FTIR spectra were recorded in the range of 4000–400 cm^−1^, with 64 scans at a resolution of 4 cm^−1^. The processing software was OMNIC 9.6.251. The shape of the bands was considered to be Gaussian and/or Lorentzian. Spectral subtractions have been performed with the OMNIC software. The analysis was also conducted on the performed preliminary synthesis in order to claim the repetitive formation pattern, and the final one was presented in the manuscript. The measurements were reproducible, with an approximate error of ±0.3 absorbance units due to both signal noise and variability in the sample preparation.

#### 2.3.3. Raman Spectroscopy

Powders were analyzed by using a confocal RAMAN LabRAM HR 800 microscope (Horiba Jobin Yvon, Japan). The samples were subjected to continuous laser radiation stemming from a diode laser at 532 nm (power: 12 mW) under an Olympus BX 41 microscope. Focus was achieved by an objective ×100 with a numerical aperture of 0.9 that provided the system with a lateral resolution of 0.72 µm and an axial resolution of 2.61 µm. Spectra were acquired using 600 and 1800 lines/mm grating, with a spectral resolution of 2 and 0.6 cm^−1^, respectively. The spectra were evaluated and decomposed with LabSpec 6 software. Additionally, spectral decompositions were performed, following the subtraction of a linear baseline, in the 1200–800 cm^−1^ domain corresponding to the ν_1_ and ν_3_ vibrational domains of phosphate species. Furthermore, to confirm the homogeneity of each sample, a DuoScanTM imaging system was applied to perform the macro-scale mapping (50 µm × 50 µm size) of the samples. The measurements were reproducible with an approximate error of 1–2 wavenumber units due to the machine signal noise and calibration preparation. 

### 2.4. Physical Characterization of the Powders

#### 2.4.1. Brunnauer–Emmet–Teller Method

Specific surface area (SSA) of the powders was determined by using the Brunnauer–Emmet–Teller method (BET) (ISO 9277:2010, QUADRASORB SI and Quadra Win) on a nitrogen adsorption–desorption isotherm obtained at −196 °C. Prior to applying the method, samples were degassed for 24 h at 25 °C (Autosorb Degasser Model AD-9) to remove any excess of moisture and vapors. The results were shown as an average value ± standard deviation from three replicates.

#### 2.4.2. Laser Granulometry

The particle size distribution of the starting α-TCP powder, 10 × SC and 100 × SC, was determined using laser diffraction granulometer (Malvern Mastersizer 3000) in dry mode. Additionally, the median size d_0.5_ of α-TCP, analogous to a volume cumulative percentage of 50%, was determined. The results were shown as an average value ± standard deviation from three replicates.

#### 2.4.3. Scanning Electron Microscopy 

The surface morphology of the final OCP particles/phases and other key transient steps/phases was visualized using a scanning electron microscope (SEM, Tescan Mira\LMU, Tescan, Czech Republic). Sample image generation was performed with secondary electrons, created at an acceleration voltage of 30 kV. Samples were secured with an electrically conductive double-sided adhesive carbon tape on a standard aluminium pin stub. Prior to the SEM measurement, samples were sputter coated with gold, using Emitech K550X (Quorum Technologies, Lewes, UK) sputter coater.

### 2.5. In Vitro Biological Studies 

#### 2.5.1. In Vitro Cytocompatibility Evaluation 

Human bone marrow-derived mesenchymal stem cells were employed to estimate the preliminary cytocompatibility of transient phases that were obtained during the synthesis. The cells were cultivated in low-glucose Dulbecco’s modified Eagle’s medium, supplemented with 15% fetal bovine serum and 1% penicillin -streptomycin at 37 °C in an incubator with a 5% CO_2_ and humidified atmosphere. The prepared samples were sterilized for three hours in 70% ethanol, washed two times with PBS, and soaked in the complete culture medium overnight. After overnight incubation, the conditioned medium was exchanged with the fresh culture medium. 

The in vitro cytocompatibility evaluation of the powders was performed in direct contact with hBMSCs. The 24-well plates were used for seeding the cells in a defined concentration (2 × 10^4^ cells/well). Seeding was completed four hours before the addition of the samples. The powders were suspended in the culture medium in the concentration of 0.5 mg/mL. The in vitro set up was cultivated for three days with a daily change of the culture medium. Morphology of the cells was monitored throughout the experiment via light microscopy. During three days, the metabolic activity of the hBMSCs was assessed every 24 h, using the fluorimetric resazurin reduction assay in accordance with the manufacturer’s instructions. The samples were incubated for four hours and successively evaluated with a spectrofluorometer (Spark, Tecan Trading AG, Mannedorf, Switzerland), using an excitation wavelength of 530 nm and an emission reading of 590 nm. 

#### 2.5.2. Cell Phenotype Morphology 

Morphology of the cells, after the three-day direct contact with the powder specimens, was evaluated via immunofluorescent imaging (IF). DAPI was used to label the cell nuclei, while Alexa Fluor 594-conjugated phalloidin was used to visualize cytoskeleton f-actin filaments. Briefly, samples were washed with PBS and fixed for 20 min with 4% paraformaldehyde. Subsequently, they were washed twice with PBS to elute the fixative and sample powder residues, and then stained with a mixture of DAPI and phalloidin in 0.5% Tween^®^-20 for one hour. Stained samples were checked using the digital light microscope (Invitrogen EVOS Floid, from Thermo Scientific, Waltham, MA, USA).

#### 2.5.3. Statistical Analysis

In all of the experiments, if applicable, each group of samples was represented by three or six replicates. Results were shown as the mean value ± standard deviation. When comparing groups of samples, analysis was performed in Prism (v8, GraphPad Software, San Diego, CA, USA), using the one-way ANOVA, Tukey’s post hoc analysis, and normal distribution Shapiro–Wilk’s test. For every comparison performed, the difference was considered as significant for *p* < 0.05.

## 3. Results

To be as certain as possible in the phase determination of the presented CaP phases, a detailed description of each characterization technique has been reported in the following sections. As the task of determining quantitatively the crystalline phase content has proven to be a tremendous challenge, the semi-quantitative analysis has been presented. The importance of a whole picture gained from multiple methods underlines the fact that assessing the phase identity (or alterations of chemical compositions—non-stoichiometry, variable hydration level, etc.) based on one single technique is not possible.

### 3.1. Phase and Composition Characterization

Prior to starting the OCP synthesis, thermal transformation of ACP (SSA 72 ± 14 m^2^/g, d_0.5_ = 75 ± 5 µm) to α-TCP was confirmed using XRD analysis (Figure S2). Characteristic maxima of α-TCP were in accordance with the reference XRD pattern provided by the ICDD (Figure S2a). FTIR and Raman spectroscopy analyses efficiently followed the XRD data, with specific α-TCP bands clearly discernible in the spectrum (Figure S2d,e). The median particle size (d_0.5_) determined by the laser diffraction granulometry was 37 ± 3 µm (Figure S2f), while the specific surface area was 8.8 ± 0.2 m^2^/g. SEM micrographs displayed the morphology of α-TCP aggregates (Figure S2b,c). The starting precursor for OCP was quantified and the powder was composed of 99% α-TCP (based on the Rietveld calculation, Profex software). Additionally, the as-synthesized low temperature α-TCP exhibits a higher SSA than the previously synthesized high temperature α-TCP [37]. Moreover, it was also higher than the α–TCP used previously for OCP formation. Indeed, the α-TCP used in the study of Bigi et al. [29] was milled and, according to the literature, SSA increases with milling, which still remained relatively low [38]. Furthermore, α-TCP production process in this study was characterized by the lower energy consumption coupled with a lower CO_2_ footprint [34,35]. 

#### 3.1.1. X-ray Diffraction

**Figure 1:**
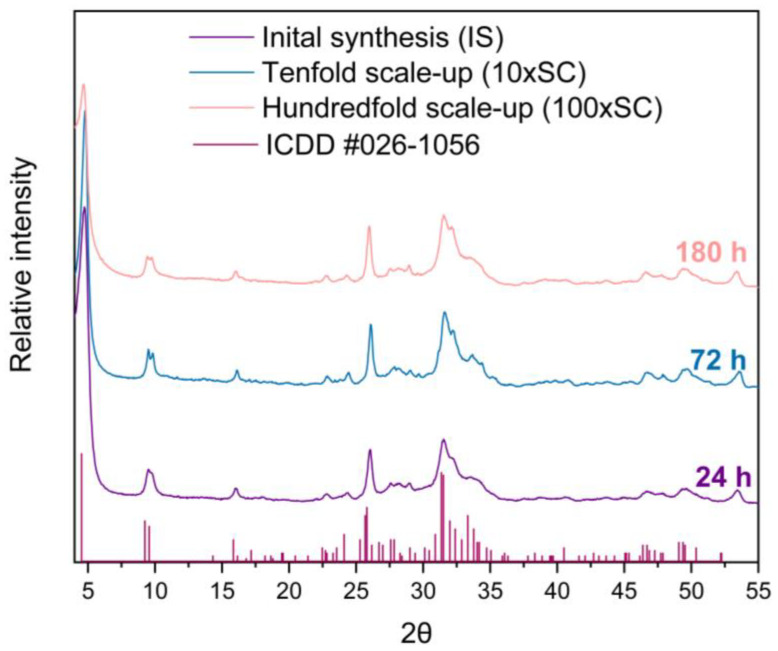
XRD patterns of final OCP powders obtained in three levels of scale-up. The reference simulated pattern (ICDD entry #026-1056) corresponds to the main maxima of the OCP triclinic phase.

The XRD pattern allows for the identification of the three main crystalline phases, with variable ratios according to the hydrolysis time: α-TCP, DCPD, and OCP. No halo or background irregularity, suggestive of an amorphous phase, were detected. The data obtained reveal that despite a constant liquid to solid ratio of the precursors, the time of the OCP synthesis has increased with the amount of the initial α-TCP used. Hence, the end of the synthesis increased with the scale-up level. The final OCP phase was attained after 24 h, 72 h, and 180 h for IS, 10 × SC and 100 × SC, respectively (Figure 1). All the X-ray diffraction peaks observed at low angles are characteristic of the OCP triclinic structure. A low angle (100) maximum at 2θ = 4.7 degrees and a doublet at 9.4 and 9.7 (200 and 010) 2θ degrees, respectively, were clearly seen (Figure 1). However, the 25–35° 2θ maxima present in the OCP structure are not very well resolved and they are also overlapping with the XRD maxima of apatite, the main impurity often reported in OCP preparation (Figures S3–S6). In reference to the intensity of the maximum at 4.7 degrees, IS (Figure S3) and 10 × SC have exhibited a higher intensity, while the overall crystallinity (observed from the peak height and resolution) appears to be the highest in 10 × SC (Figure S4).

**Figure 2:**
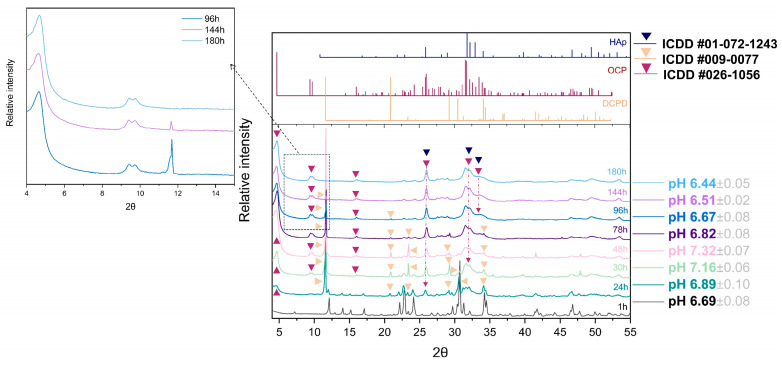
Hundredfold scale-up (100 × SC): sequence of XRD patterns illustrating the gradual transition from α-TCP phase via DCPD to OCP phase at pivotal time points chosen due to the prevalent, visually noticed change, accompanied by the fluctuation in pH values.

A detailed progression of the OCP phase formation has been followed as a function of time. Due to the great number of samples (Table S1), analysis of the vital time points of the 100 × SC synthesis (1 h, 24 h, 48 h, 78 h, 96 h, 144 h, and 180 h) and final OCP phases will be shown in the main manuscript, while other time points can be found in the SM, as indicated in Table S1. XRD patterns have shown the gradual transition from the α-TCP phase via DCPD to the OCP phase, while their presence was shifting in dependence to the observed time point (Figure 2). Visually noticed changes were accompanied by the fluctuation in pH values (Figure 2). The same phase transformation pattern has been observed across all scale-up levels, and evolution of the crystalline phase went from the α-TCP as a starting point to the mix of α-TCP, DCPD, and OCP and finally to the OCP phase. As it was mentioned, the apatite phase has a strong resemblance to OCP, thus the corresponding reference patterns have been added throughout the graphs.

**Figure 3:**
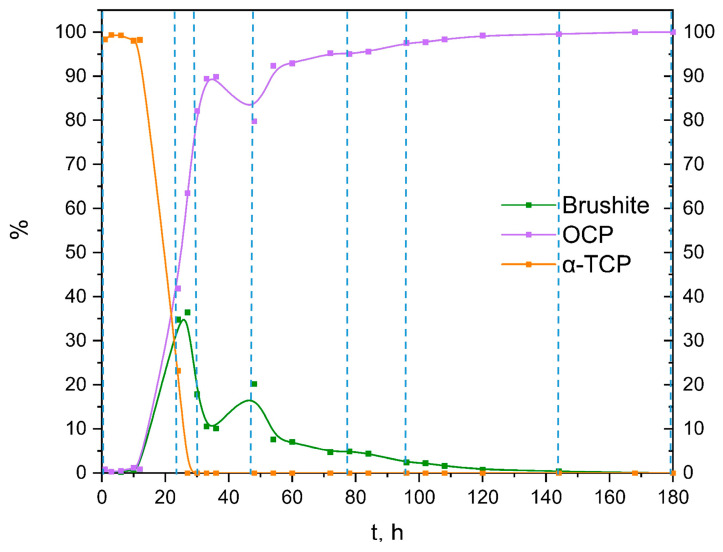
Quantitative analysis of the crystalline phases involved in the α-TCP transformation to OCP, based on XRD data from 100 × SC (after Rietveld refinement using Profex software [36]); vertical lines present the time points analyzed in detail in the main manuscript.

By using the Rietveld refinement, the progression of the 100 × SC phases formation based on the XRD method has been quantified and displayed in Figure 3 and in Section 3.2 (together with SEM micrographs in the Section 3.2). The progression of the time of the CaP phase interchange was an evolution from ~100% of α-TCP (in the first hour) to ~37% of DCPD and ~63% of OCP (at 27 h) and ended with ~100% of OCP. The OCP ratio versus the reaction time of IS and 10 × SC, authenticated via XRD patterns, can be found in the Supplementary Materials (Figures S3 and S4, respectively). Moreover, additional kinetic time points of the 100 × SC OCP formation can be seen in Figures S5 and S6.

#### 3.1.2. Fourier-Transform Infrared Spectroscopy and Raman Spectroscopy

**Figure 4:**
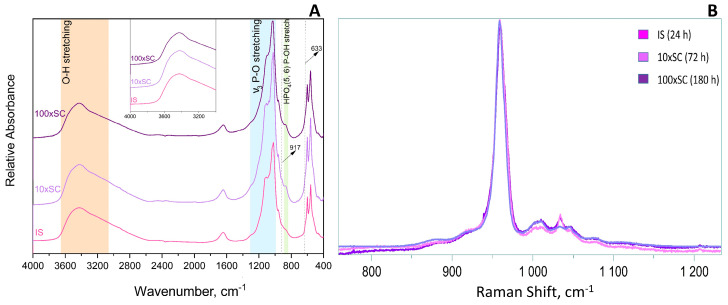
FTIR (**A**) and Raman (**B**) spectra of final OCP phases obtained in three levels of scale-up.

**Figure 5:**
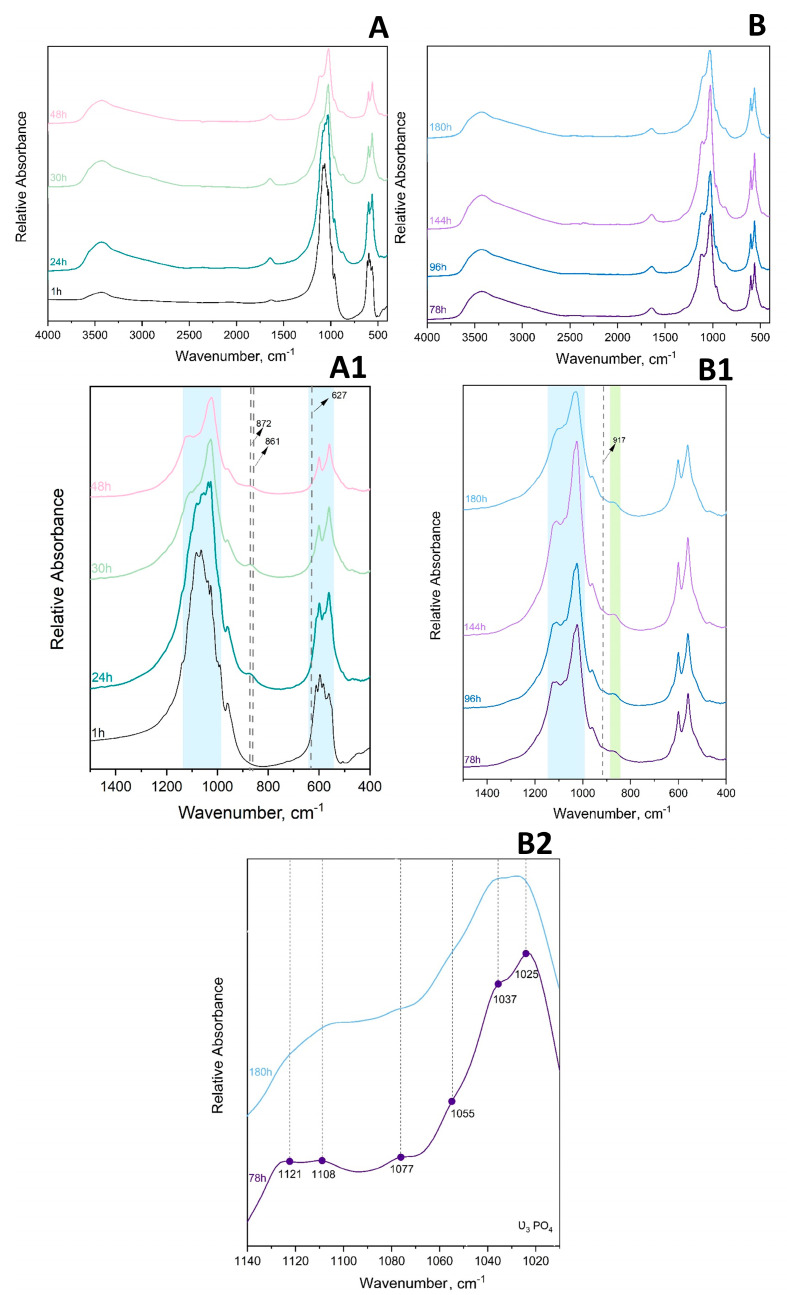
FTIR (**A**,**A1**) absorbance spectrum of 1 h–48 h 100 × SC and (**B**,**B1**,**B2**) of 78 h–180 h 100 × SC. Green shadowed area is marking the narrow range of HPO_4_ (5, 6) P–OH stretch and blue shadowed area corresponds to PO_4_ stretching.

The ν_1_ P–O stretching mode clearly displayed the strongest lines expected for the OCP phase in Raman (Figure 4B), whereas in FTIR it was the ν_3_ P–O stretching mode at 1300–1000 cm^−1^ (Figure 4A). In addition, characteristic vibrations of HPO_4_^2−^, which despite being weak, differentiate the OCP from stoichiometric HAp and were clearly seen at 917, 875, 1007, and 1295 cm^−1^ (blue and green shading, Figure 4A). The OCP samples often show unexpected OH^−^ vibration modes at 3570 cm^−1^ (stretching mode) and 633 cm^−1^ (libration mode) related to the evolution of the structure towards the more stable HAp [39]. The assignment of the line at 633 cm^−1^ to HAp impurities in OCP has been discussed and, according to Fowler, could correspond also to the water libration line of OCP [40]. A very faint OH^−^ stretching line can be distinguished as a shoulder on the strong H_2_O stretching line in the long lasting 10 × SC and 100 × SC syntheses (orange shading in Figure 4A). Furthermore, it can be noticed that 100 × SC absorbance bands (Figure 4A and Figure 5B1,B2), in the region from 1130–1020 cm^−1^ (blue shading Figure 4A), seem to be somewhat less resolved, which could signal a lower crystallinity of OCP in comparison to lower scale-ups (IS and 10 × SC) [41].

##### FTIR Spectroscopy

The main observations in the IR spectrum were as following: within the first 12 h of 100 × SC, the most intense bands of α-TCP in the PO_4_ region are barely observed (1300–900 cm^−1^ domain and 700–500 cm^−1^ domain). Almost no spectra changes in the period between 33 h and 180 h period were noticed (Figure 5A–B1, Figures S10 and S11). The ν_3_ stretching mode of PO_4_^3−^ and HPO_4_^2−^, at 1077 cm^−1^, 1093 cm^−1^, and 1121 cm^−1^, with the faint but characteristic line of OCP HPO_4_(6) [P-(OH)], stretch at 917 cm^−1^ and O–H in-plane bending around 1295 cm^−1^ were clearly seen at the 78 h time point (Figure 5B2). The H_2_O stretching mode of structural water, allocated to DCPD (~3500 cm^−1^), was observed at 24 h of 10 × SC (Figure S9), as well as in the initial synthesis at 10 h and 12 h (Figure S8) [42,43]. The DCPD characteristic absorbance band at 790 cm^−1^, which corresponds to a broad libration water line, has been noticed at 24 h 10 × SC (Figure S9).

To enhance the sensitivity of FTIR spectroscopy, spectral subtractions have been performed to remove overlapping absorbance, improve absorbance bands of interest, and/or to try to further discern the DCPD bands [44]. Following this approach, we have subtracted the absorbance bands of the final 100 × SC OCP phase 180 h and 100 × SC 78 h time point (Figure S12) and between the 78 h/48 h time point of 100 × SC (Figure S12). The main reasoning was due to the previously mentioned observation that even at the 78 h point, OCP was well transformed. Furthermore, the spectral subtraction has been additionally applied on the 10 × SC 72 h/24 h-time point (Figure S13) to enhance the DCPD band vibrations even more. The spectrum of the 100 × SC 180 h/78 h subtraction has not revealed a significant difference other than the more pronounced bands at 2900 cm^−1^ (connected with CH stretching of organic impurities possibly generated by wear particles resulting in a long stirring time) [43]. The subtraction result of 78 h/48 h and 10 × SC 72 h/24 h had substantially more DCPD bands within a very broad 3000 cm^−1^ region and was also masked in the ν_1,3_ PO_4_ region. Moreover, for the 10 × SC 72 h/24 h, a very expressed broad band at 790 cm^−1^ corresponding to the out-of-plane bending of the HPO_4_^2−^ in DCPD was revealed via subtraction.

##### Raman Spectroscopy

**Figure 6:**
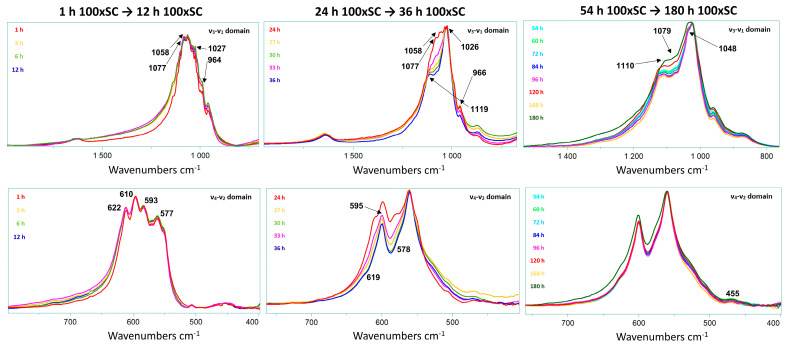
Raman spectra of the OCP formation as a function of time in 100 × SC. The graph displays a close up of ν_1_–ν_4_ stretching mode of the P–O vibration. Assignment of the highlighted bands can be found in Table S2.

Analogous to FTIR, a clear transition from α-TCP powder to a sample where OCP is in majority was noticed in the first 24 h of 100 × SC (Figure 6). The bands with a strong intensity were mostly located in the region between 900–1000 cm^−1^, which corresponds to the ν_3_ triple-degenerate asymmetric P–O stretching mode and partly to the ν_1_ symmetric P–O stretching vibration. After the 30 h time point, the presence of the characteristic α-TCP band arrangements evolved to OCP (Figure 6). The 964 cm^−1^, 976 cm^−1^, 984 cm^−1^, and 998 cm^−1^ bands have vanished, and the most prominent band at 958 cm^−1^ also present in OCP has remained. Over the time period, this broad band evolved into a massif, composed of two more marked bands at 965 and 958 cm^−1^ that were characteristic of OCP (Figure S18). The low resolution of this massif could indicate a poorly crystallized OCP phase. Furthermore, the second derivative of the Raman spectra did not reveal the presence of the apatite phase, which could attest to OCP, although the detection of a poorly crystalline apatite would not be easy to detect in this system with broad lines.

In order to determine the homogeneity of the samples, Raman analyses carried out in micro spot were confronted with an analysis in macro spot (having a beam size of 50 × 50 µm (duoscan)) for each time point in 100 × SC. For the beginning time points of OCP formation (up to 12 h), the samples presented a homogeneous composition of α-TCP, but above this time point and up to 48 h, several signatures comprising different phase ratios were observed (Figure S16). 

#### 3.1.3. Specific Surface Area and Particle Size

Specific surface area results have been reported in the Table 1. High SSA values of all the final OCP phases may have an impact on the physicochemical and biological reactivity of the material.

**Figure 7:**
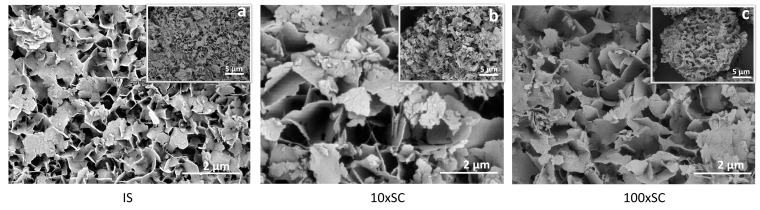
SEM micrographs of the final OCP phases (scale bar 5 µm (top right) and 2 µm (bottom right)): (**a**) initial synthesis (24 h time point), (**b**) tenfold scale-up (72 h time point), and (**c**) hundredfold scale-up (180 h time point).

**Figure 8:**
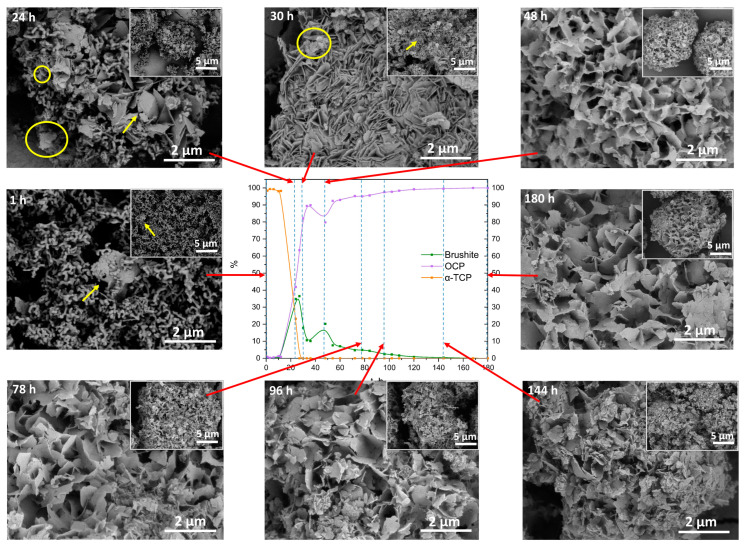
Scanning electron micrographs of α-TCP transformation to OCP (100 × SC), observed at specific time points (1 h–180 h) indicated on each SEM image (scale bar 5 µm and 2 µm) and connected (red arrow) to the according point (dashed line) in the phase content diagram based on the XRD quantitative data. Yellow arrow indicates possible DCPD crystals, while the yellow circle is marking the potential OCP plates.

Particle size distributions have been presented in Figure S21. The starting α-TCP precursor had a monomodal curve (Figure S2f), and every transformation time point analyzed exhibited bimodal curves. The final OCP phases in both scale-up levels had the smallest primary particle size in the range of 5–25 µm, equaling to a ~5% volume fraction. However, the presence of the secondary distribution in the range of 150–500 µm indicated that the primary particles were unevenly agglomerated. Considering that the measurement has been conducted on the dry powder samples, the release of pressure applied during the analysis might not have been strong enough to separate certain agglomerates. Since the size of the individual OCP crystals appears to be smaller than several μm (Figure 7, Figure 8 and Figure S21), small, loosely aggregated OCP plate-like particles could be denoted as nano-submicro OCP. Moreover, high SSA is a strong indicator of submicro size, at least in one dimension.

### 3.2. Morphology Characterization

The final OCP phases exhibited plate-like morphology, with crystals having uneven edges and being approximately 100-nm thick (Figure 7). The plates seemed to be 1–3 μm in width, with smaller chipped plates being one over the other, indicating that OCP plates grew over the larger DCPD plates. In all scale-up levels, agglomeration took place and thin plate-like crystals formed loosely packed agglomerates (Figure 7, Figure 8, Figures S19 and S20).

When the individual follow-up time points were observed via SEM, a clear change in the phases could be seen. After 1 h of 100 × SC, the sample contained thin thread-like crystals of α-TCP, however, small needle- or plate-like crystals started to appear within the α-TCP aggregates (Figure 8, 1 h). As the synthesis progressed, at the 24 h and 30 h time points (Figure 8, 24 h, 30 h), a gradual increase to larger and thicker plate-like particles was observed. It could be concluded that the larger and substantially more massive plates at those time points were attributed to DCPD (marked with an arrow) and smaller thinner plate-like crystals of OCP started to form (marked with a circle). However, this is hard to differentiate due to the high morphological similarity of the two crystals/phases. The same trend was observed with the initial time points of IS (Figure S19) and 10 × SC (Figure S20). Throughout the rest of the synthesis, a roughly uniform appearance of the crystals remained. Crystals stayed in the same plate-like shape, with noticed fragmentation of the larger particles and continuous presence of the spherical aggregates.

### 3.3. In Vitro Biological Assays

#### 3.3.1. Cell Viability Assessment

**Figure 9:**
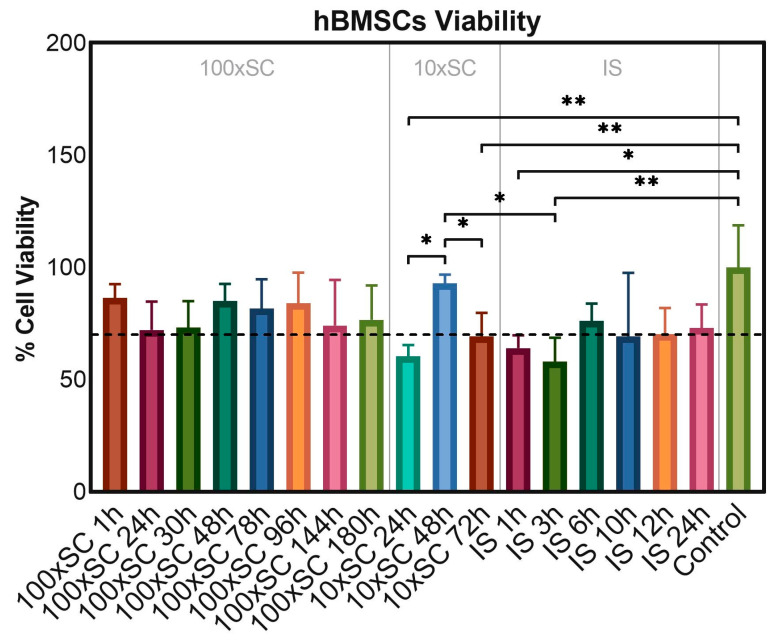
In vitro cell viability of the final and the transient powders after three days of incubation in direct contact with hBMSCs. ‘Control’ series represented cells cultivated on polystyrene without CaP samples. Error bars represent the mean ± standard deviation of three replicates. Multiple comparisons were performed on relative fluorescent units via one-way ANOVA with Tukey’s correction; significant differences between groups of samples were indicated with asterisks (* for *p* ≤ 0.05, ** for *p* ≤ 0.01).

To evaluate the cytotoxicity of the final and the transient powders, samples were incubated in direct contact with hBMSCs for 3 days at the concentration of 0.5 mg/mL. Samples were considered non-compatible with hBMSCs, if the cell viability was lower than 70% following ISO 10993-5:2009(E) [45]. The resazurin reduction fluorimetric assay showed a decrease in the metabolic activity of cells in contact with powders in comparison to cells on polystyrene. However, on the third day of cultivation, only five types of seventeen tested samples—10 × SC 24 h, 10 × SC 72 h, IS 1 h, IS 3 h, and IS 10 h—resulted in the hBMSC metabolic activity being slightly lower than the 70% threshold, while the other time points of each scale-up were above the threshold and thus were considered cytocompatible (Figure 9). Compared to the control group, represented by the cells grown on polystyrene, three out of five non-cytocompatible samples led to a significant decrease in the metabolic activity of hBMSCs (10 × SC 24 h, IS 1 h, and IS 3 h).

#### 3.3.2. Cell Phenotype Morphology

**Figure 10:**
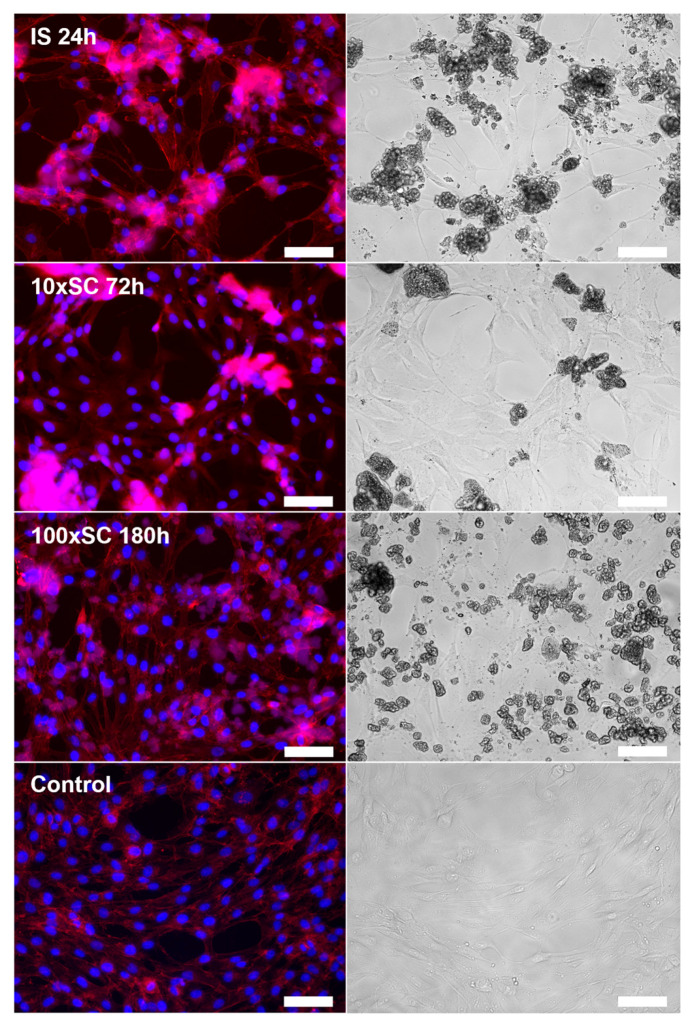
hBMSCs morphology on the third day of cultivation in direct contact with final OCP samples (IS, 10 × SC and 100 × SC) in the concentration of 0.5 mg/mL. Control represented cells on polystyrene. Immunofluorescent (**left** column) and bright-field (**right** column) microscopy. Image bar scale: 125 µm.

Confirming the results obtained in the metabolic activity assay, microscopy revealed a normal spread, attachment, and morphology of the cells incubated in direct contact with the 100 × SC samples (Figure S22 and Figure 10) when compared to cells cultivated on polystyrene (Figure S22, ‘Control’). Samples from the group of tenfold synthesis (10 × SC) demonstrated an uneven spread and lower confluence of the cells in the observation area, and more importantly, the presence of a relatively large amount of powder particles (Figure S23). The most cytotoxic result within this scale-up, in terms of the resazurin reduction assay (IS 3 h sample series—58 ± 10.6% cell viability), demonstrated an even spread of slightly larger agglomerates, while in the comparably toxic series of IS 1 h (64 ± 5.7% cell viability) the agglomerates were smaller with a same even spread. The morphology of the cells in the group was not altered.

## 4. Discussion

### 4.1. Phase and Composition Characterization

#### 4.1.1. X-ray Diffraction

In general, the OCP formation via the hydrolysis of α-TCP is represented by the following chemical equation [41].

3 Ca_3_(PO_4_)_2_ + 7 H_2_O → Ca_8_(HPO_4_)_2_(PO_4_)_4_·5H_2_O + Ca(OH)_2_ (1)

This global reaction induces an increase in the solution pH, related to the proton uptake by PO_4_^3−^ groups in the OCP phase and a corresponding release of soluble Ca(OH)_2_. As it was seen in Section 3.1.1, the time of the OCP synthesis has increased from the initial 24 h to 72 h and to 180 h for IS, 10 × SC and 100 × SC, respectively (Figure 1). The X-ray diffraction maximum that was observed at low angles is characteristic of the OCP triclinic structure (4.7 2θ degrees). However, this most intense and very specific peak of the OCP structure is superimposed to the X-ray diffusion background and not easily accessible. In addition, one must take into consideration that due to the plate-like morphology of OCP, preferential orientations can appear, which strongly affect this peak’s intensity. This is why different authors often consider the doublet at 9.4 and 9.7 2θ degrees as a factual representative of the OCP triclinic phase [9,28,46,47]. As it was mentioned before, the same crystalline phase evolution has been seen across all scale-up levels (α-TCP, mix of α-TCP, DCPD and OCP, and finally OCP). Thus, the XRD patterns of samples collected at different synthesis time points clearly show that global reaction one, describing the direct conversion of α-TCP into OCP, is inadequate, as the DCPD has been identified as an intermediary phase during this reaction (Figure 2 and Figures S3–S6). Within the suspension medium, the pH variations are sensitive indicators of the chemical reactions involved, which allow us to clarify the different steps of this conversion. Hence, several steps were distinguished as follows.

##### Dissolution 

At the beginning, the 0.0016 M phosphoric acid solution pH was 2.80 ± 0.15. At the addition of the α-TCP powder, a rapid increase in pH was observed, which stabilized at 6.69 ± 0.08 at 1 h. This first event corresponded to the fast dissolution of α-TCP according to reaction two (proposed for a 6.7 end pH):

Ca_3_(PO_4_)_2_ + 3 H_2_O → 3 Ca^2+^ + H_2_PO_4_^−^ + HPO_4_^2−^ + 3 OH^−^ (2)

This step, implying a release of OH^−^, puts the reaction medium at a pH close to neutrality, thus corresponding to the buffering zone of orthophosphate anions (Figure S7).

##### Precipitation

Considering the solubility of α-TCP (and to some extent, the initial acidic dissolution, followed by a strong pH variation towards alkaline medium), the solution obtained is highly supersaturated with respect to OCP, DCPD, and also HAp. A strong advantage of OCP and DCPD is that they show a better ability to nucleate and grow (higher crystallization rates) than HAp [48,49,50]. The formation of these phases from the ions in the solution corresponds to the release of protons and the decrease in pH.

##### Growth Step of DCPD and OCP Phases

The α-TCP to DCPD conversion appeared to be much faster than that of α-TCP to OCP, which was in accordance with the crystal growth rate of these two compounds [10]. The reaction of DCPD growth from the α-TCP hydrolysis is as follows:

Ca_3_(PO_4_)_2_ + 3 H_2_O → 3 Ca^2+^ + H_2_PO_4_^−^ + HPO_4_^2−^ + 3 OH^−^ (3)

implying that, like for OCP formation (Equation (1)), an alkalinization of the solution and these two reactions (Equations (1) and (3)) raise the pH up to 7.32 ± 0.07 (Figure 2). At the onset of the highest pH value (48 h 100 × SC), the strongest X-ray diffraction peak of DCPD, the (020) reflection, becomes noticeably more intense than any other diffraction peak maximum (Figure 2).

##### DCPD to OCP Conversion Step

When α-TCP has been completely dissolved, DCPD became the most soluble phase of the system and also the precursor for the conversion into OCP, which corresponds to the historically most used preparation method of OCP (Equation (4)) [39]:

8 CaHPO_4_·2H_2_O → Ca_8_(PO_4_)_4_(HPO_4_)_2_·5H_2_O + 2 H_3_PO_4_ + 11 H_2_O (4)

This reaction results in a release of protons in the solution, which has been attenuated in the system due to the buffering properties of the present phosphate medium, resulting with the final pH of 6.44 ± 0.05. 

#### 4.1.2. Fourier-Transform Infrared Spectroscopy and Raman Spectroscopy

In order to delve deeper into the analysis of the α-TCP to OCP formation pathway and to get the information on the phases at the molecular level, FTIR and Raman spectroscopy have been employed as the chosen techniques. In addition, as the unit cell of OCP contains two HPO_4_ crystallographic sites, labelled HPO_4_(5) and HPO_4_(6), their P–(OH) stretch and OH in-plane bend cannot be distinguished via the XRD method. Therefore, the IR and Raman spectra can help in discerning the progressive hydrolysis. The spectra of the final OCP phases (Figure 4) were in agreement with the XRD patterns and with the data provided in the literature (Table S2). By observing the analysis provided via the FTIR technique, it can be seen that the analysis of the phase transformation between the first occurrence of the OCP phase and the end of OCP formation does not fit well with the XRD results (24 h for 100 × SC and 10 × SC, 10 h for IS). The extreme anisotropy of the DCPD crystals or extreme ordering of them have resulted in a relatively high phase presence in XRD (up to ~36% at 27 h for 100 × SC), but in the FTIR and Raman spectra DCPD has been barely noticed. The α-TCP phase appears also difficult to detect in mixtures due to the profusion of expected lines associated with a dispersion of their intensities. As it is difficult to monitor the progression of OCP and its potential evolution with time via FT-IR spectroscopy, the main focus will be placed on the most important lines that separate it from both the transitional phases and from the possible presence of calcium-deficient apatite (CDHAp) (which can also be obtained after ageing). The spectral data throughout the literature have suggested that the main difference between the spectra of OCP and the apatite phase reside in HPO_4_^2−^ vibration lines [3,40,51,52,53]. Thus, the assignment of HPO_4_^2−^ lines bending and O–H stretching modes with bands connected with H_2_O will be highlighted. 

##### Assignment of HPO_4_^2−^ Ion bonds

By observing a single OCP unit cell, six phosphate groups can be differentiated (P1–P6). P1–P4 can be found within the apatite-like layer, distributed on two triangular sets (two Ca^2+^ and two PO_4_^3−^ per each set), while the remaining two (P5, P6) are serving as a connection bridge between the two asymmetrical cell units [52]. During the phase transformation from OCP into CDHAp, a decrease in HPO_4_(6) happens first (in regard to HPO_4_(5) which is close to the vibration line of an apatitic HPO_4_). The most significant HPO_4_ lines assigned to OCP are: 1295 cm^−1^ assigned to the OH in-plane deformation mode and 917 cm^−1^ to the P(6)–(OH) stretch of a strongly hydrogen-bonded HPO_4_^2−^ ion [40,52,53]. For all the assignments see Figure 5 and Figures S8–S11.

##### O–H Stretching Modes and Bands Connected with H_2_O

Even though the OCP spectral range between 2500 and 3700 cm^−1^ has been portrayed by a very broad absorbance band (with an overtone and combination bands present), it shows an important distinction from apatite. The lack of a recognizable 3572 cm^−1^ OH^−^ band for HAp is one of the indicators of a pure unaltered OCP formation (or at least with minimum HAp presence) [40]. Another rather characteristic OH^−^ line at 633 cm^−1^ is assigned to the OH^−^ libration movement in HAp. A line is also found in this range (627 cm^−1^) as a shoulder in OCP, which has been assigned by Fowler et al. to a libration movement of the H_2_O(4) water molecule of OCP. Thus, only the OH^−^ stretching line at 3572 cm^−1^ is a marker of HAp formation, which has not been detected.

The small presence of DCPD noticed in the spectrum recorded for 100 × SC could be attributed to the heterogeneity of the sample. As DCPD crystals tend to be larger in size, even a small number of them in the whole batch could be recognized using the XRD method. Nevertheless, for FTIR analysis (Figure 5 and Figures S8–S11), a considerably smaller amount of specimen is used, hence a probability of missing the crystals in the composition is possible. 

Similar to FTIR, Raman band vibrations are also closely packed and most of the time are overlapping, hence all of them have been disclosed in Table S2, while the most important ones were discussed in Section 3.1.2. After observing the attained bands from the micro and macro spot in Raman, the differences between these two measurement modes could indicate that the formation and/or dissolution of the phases were intimately linked to the size of the grains (or aggregates) of the starting powders. From 100 × SC 24 h, an increase in the band at 959 cm^−1^ and a widening of the main massif has been noted, authenticating the formation of OCP. However, a different proportion of the present phases was observed between the two measurement modes. Once the sample at 100 × SC 27 h has been analyzed with duoscan, a change in the band at 962 cm^−1^ was observed. This, together with the “red shift” of the massif, when compared to the spectrum obtained in micro spot mode, could attest to the presence of apatite or/and a greater proportion of α-TCP (Figure S17). For the analysis carried out in the micro spot mode, an additional band was observed at 949 cm^−1^ (for 27 h), which could be attributed to an amorphous TCP phase, as well as an additional band at 970 cm^−1^, which has not been attributed. From 100 × SC 48 h until the end of the synthesis, a single profile has been observed between the micro spot and macro spot. A total absence of the band at 917 cm^−1^ can be noted, which has pointed to the end of α-TCP presence. In addition, the large mass may be associated to the amorphous phase(s) and/or related to the convolution of several crystalline phases composed of the main band at 959 cm^−1^ and the shoulder at 966 cm^−1^, associated with ν_1_PO_4_ of OCP, but also to the HPO_4_ environments at 870 and 917 cm^−1^. Nevertheless, the uncertainty of containing characteristic bands of a more or less well-crystallized apatite or an amorphous phase (TCP) has to be thought of.

However, similar to the FTIR spectra, only a small number of vibration bands corresponding to DCPD were observed in the Raman spectra. In the spectrum of 10 × SC, the specific band at 986 cm^−1^ was noticed (Figure S15). At 24 h, the band’s presence was pronounced, but still not as strong, whereas at 48 h the band in the PO_4_ stretching region was significantly stronger and quite easily discernible. The same position of the band can be seen also in the IS 12 h time point (Figure S14). Alongside the aforementioned band, the intense vibration of the HPO_4_ ion at 877 cm^−1^ in 10 × SC is indicative of the presence of DCPD in the phase mixture, but at the same time, the HPO_4_ ion of OCP vibrates at the same frequency. Furthermore, it would seem that in the kinetics of the formation of the OCP, the time points of 10 h and 12 h of IS are opposite. This is, on one hand, due to the heterogeneity of the mixtures, but also due to the differences in the scan size of each entity (micro spot of the laser).

### 4.2. Morphology Characterization

OCP crystals grow along (010) and (100) faces, with [001] persisting as the dominant growth direction, commonly having the appearance of thin plate-like crystals [3,39,51]. DCPD has a similar morphology, but the crystals are bigger and heftier (width between 5 and 10 μm, with possible elongation to 70–80 μm) [26]. The formation of the specific OCP crystals is considered to be connected to the Hartman–Perdok theory of periodic bond chains [54]. The theory suggests that a continuous path of strong bonds within the crystal structure has a portion of the lattice which is cut by a certain face (hkl). In the case of OCP, the plates grow in the [001] direction, with the largest face, 100, while in width the predominant faces are 110 and 010. Numerous 100 interconnections, at low synthesis temperatures, lead to the formation of spherical aggregates looking like sand roses. At the same time, as we described in Section 3.1.1, while the dissolution of α-TCP is ongoing, it is being followed by the growth step of DCPD, and from DCPD, transformation to the OCP phase transpires. Hence, multiple factors influence the morphology of the particles.

### 4.3. In Vitro Biological Assays

The main goal behind doing the in vitro biological assay was to establish the cytocompatibility behind each mix of the CaP phases throughout the reaction scale-up. In other words, as there was no record of the OCP synthesis scale-up, it was highly necessary to check if the initial cytocompatibility of OCP remained. Furthermore, as it was possible to scale up the initial synthesis from 100 mg to 10 g, the next step in the research would be to try to scale up even further. However, an additional scale-up would imply that in order to obtain OCP as a single phase, the reaction would last even longer. Thus, several questions need to be answered: firstly, if we scaled up the reaction 100 times, is the final product still as cytocompatible as the original synthesis product? What will be the cell response to the mixture of CaP phases during the transition? Finally, would a certain phase ratio demonstrate better cytocompatibility with cells than pure OCP itself? If a mix of OCP, DCPD, and/or α-TCP would show a desirable outcome when subjected to a direct contact with cells in vitro, would it be safe (or even more preferable) to stop the next level of scale-up at a specific time?

Presented results in Section 3.3 have confirmed that throughout the entire pathway of OCP formation (IS, 10 × SC and 100 × SC) the obtained products are safe to use in biological assays. Furthermore, if the time points between 48 h and 96 h of the 100 × SC scale-up were taken into consideration and the reaction was stopped at a point of having 80% of OCP, the results would still yield > 80% of cell metabolic activity. However, in order to be certain in such a claim more studies are needed, followed up with assessment of the pro-osteogenic efficacy at each time point. Based on the XRD quantitative data calculated with Profex, at IS 1 h and IS 3 h CaP the phase content was >92% α-TCP, <8% OCP, and for 10 × SC 24 h the phase content was ~25%, ~45% OCP, and ~29% DCPD. It is not completely sure why the starting time points of each scale-up exhibited the lowest cell viability. The authors believe it was connected with the pH change that would happen during the exposure of the powders to the cell medium. As eventually each phase is expected to transform to CDHAp or OCP (as an intermediate phase), the transformation from the starting mix of phases (α-TCP, DCPD present) would have created a more alkaline environment due to the release of OH^−^ ions (see Section 3.1.1). An additional factor that could influence the percentage of viable cells is the size of the agglomerate that is being formed in the direct contact with the cells.

To additionally corroborate the phase influence on the cells and to monitor the cell morphology throughout the incubation period, bright-field microscopy was used. On the third day of hBMSCs cultivation with the samples, visual analysis was complemented with immunofluorescent staining. All kinetic time points (IS, 10 × SC and 100 × SC) have been analyzed, and the cell morphology after the contact with final OCP phases has been shown in Figure 10. Considering the procedure of staining, which includes the multiple rinsing of cells with PBS, the observation of particles points to either a strong attachment of the material to the cell layer or to their internalization into hBMSCs. The presence of powder particles was also observed in the culture plate wells with the initial synthesis (Figure S24). However, the amount and size of the particles and/or agglomerates in the observation area were poorly correlated with the previously described viability rate. By following the cell morphology, within the scope of this research we found no clear connection between the amount and size of the CaP powder particles and the previously described cell viability rate. However, the morphology of the cells in all sample groups was not altered. Further studies are needed in order to assess whether there is a correlation between the tested powders and possible CaP particle internalization within the hBMSCs.

## 5. Conclusions

Within the present study, OCP was synthesized from α-TCP (prepared from ACP) at room temperature via the hydrolysis method and the process was for the first time successfully scaled-up (tenfold and hundredfold). By following the formation kinetics at multiple time points, the gradual transition from the α-TCP phase via DCPD to the OCP phase was established and characterized using a multi-technique approach. 

The α-TCP–DCPD–OCP transformation chemistry encompassed the dissolution step, precipitation step, growth of DCPD/OCP, and the step of DCPD conversion into OCP. Previously well-recognized phase fingerprints confirmed the final OCP phases: maxima at 4.7 2θ degrees and the doublet at 9.4 and 9.7 2θ degrees; IR assignment of HPO_4_^2−^ ion at 524 cm^−1^, 1295 cm^−1^, and 917 cm^−1^, together with the lack of a recognizable 3572 cm^−1^ O–H band for Hap and the ν_1_ P–O stretching mode in Raman. Due to the phase interweaving (α-TCP–DCPD–OCP), the inhomogeneity of the samples was seen in Raman in the beginning stages of the synthesis when macro spot analysis was applied. The morphology of the OCP samples in all scale-up levels showed the characteristic plate-like structure. Moreover, in order to test the biocompatibility of not only final OCP phases, but also all the transient phases, in vitro cell toxicity analyses were performed and no cytotoxic effects were observed for 12 out of 17 tested samples. Cell phenotype morphology showed possible CaP particle internalization within the cells when hBMSCs were subjected to direct contact with the powders. This phenomenon, for the first time, indicated that the upscaling of the method did not negatively influence the cell viability. Furthermore, it could denote the proof of concept for the potential usage of the DCPD/OCP phase mixture (presented in the intermediary time points) for in vitro and in vivo purposes.

## Figures and Tables

**Table 1:** Specific surface area of the starting precursor and the final OCP phases at all levels of scale-up.

	α-TCP	OCP, IS	OCP, 10 × SC	OCP, 100 × SC
SSA, m^2^/g	9.0 ± 0.6	53 ± 6	66 ± 5	63 ± 8

## Data Availability

The data presented in this study are available on request from the corresponding author. The data are not publicly available due to the connected study.

## Supplementary Materials

The following supporting information can be downloaded at: https://www.mdpi.com/article/10.3390/biom13030462/s1. Figure S1. OCP formation from α-TCP suspension—follow-up methodology; Figure S2. Characterization of the starting α-TCP powder by (a) XRD, (b,c) SEM, (d) FTIR and (e) Raman spectroscopy, and (f) laser granulometry; Figure S3. XRD pattern of the specimens collected at different time points during 24 h of IS, with principal phase reference XRD patterns attained from ICDD; Figure S4. XRD pattern of the specimens collected at different time points during 72 h of 10 × SC, with principal phase reference XRD patterns attained from ICDD; Figure S5. XRD pattern of the specimens collected at different time points from 3 to 54 h during 100 × SC and principal phase reference XRD patterns attained from ICDD; Figure S6. XRD pattern of the specimens collected from 60 to 168 h during 100 × SC, and principal phase reference XRD patterns attained from ICDD; Figure S7. Speciation of orthophosphate species according to the pH. Drawing adopted from Wikipedia; Figure S8. FTIR absorbance spectrum of specimens collected at different time points during the 24 h of IS; Figure S9. FTIR absorbance spectrum of powders collected at 24, 48 and 72 h of 10 × SC; Figure S10. FTIR absorbance spectrum of powders collected at different time points during 36 h of 100 × SC; Figure S11. FTIR absorbance spectrum of specimens collected at different time points during the period from 54 h–168 h of 100 × SC; Figure S12. FTIR subtraction absorbance band between 100 × SC 78 h and 180 h samples (upper row) and FTIR subtraction absorbance band between 100 × SC 48 h and 78 h samples; Figure S13. FTIR subtraction absorbance band between 10 × SC 24 h and 72 h samples; Figure S14. Raman spectra of the powders collected at different time points during initial synthesis (IS); Figure S15. Raman spectra of the powder evolution as a function of time in 10 × SC synthesis; Figure S16. Raman spectra comparison between micro (light color) and macro spot (dark color) of the powders collected during 100 × SC; Figure S17. Raman spectra comparison between micro (light color) and macro spot (dark color) of the powders collected after 24 h and 27 h during 100 × SC; Figure S18. Raman spectra of final OCP phases obtained in three levels of scale-up and the representation of the second derivative of the most prominent band at 958 cm^−1^; Figure S19. Scanning electron micrographs of α-TCP transformation to OCP (initial synthesis), observed at specific time points indicated below each SEM image; Figure S20. Scanning electron micrographs of α-TCP transformation to OCP (10 × SC), observed at specific time points indicated below each SEM image; Figure S21. Particle size distribution and SEM micrographs of powders collected at different time points during 10 × SC and 100 × SC, showing the presence of agglomerated crystals; Figure S22. hBMSCs morphology on the third day of cultivation in direct contact with hundredfold synthesis OCP samples in the concentration of 0.5 mg/mL; Figure S23. hBMSCs morphology on the third day of cultivation in direct contact with ten-fold synthesis OCP samples in the concentration of 0.5 mg/mL. *Control* represents cells on polystyrene. Immunofluorescent (DAPI—blue, phalloidin—red) and bright-field microscopy; Figure S24. hBMSCs morphology on the third day of cultivation in direct contact with initial synthesis OCP samples in the concentration of 0.5 mg/mL; Table S1. List of all the analyzed samples collected at different time points and their presentation within the main manuscript and the supplementary information (SI); Table S2. FTIR and Raman band assignments for OCP, α-TCP and DCPD.
