# Supplementary material for: Exploring the Formation Kinetics of Octacalcium Phosphate from Alpha-Tricalcium Phosphate: Synthesis Scale-Up, Determination of Transient Phases, Their Morphology and Biocompatibility
Source: Biomolecules. 2023 Mar 2;13(3):462. doi: 10.3390/biom13030462 (PMC10046208; doi:10.3390/biom13030462)
Supplement: Supplementary file 1 [file biomolecules-13-00462-s001.zip › biomolecules-2180517-supplementary.pdf]

SUPPLEMENTARY MATERIAL

Exploring the formation kinetics of octacalcium phosphate from alpha-tricalcium phosphate: synthesis scale-up, determination of transient phases, their morphology and biocompatibility

Ilijana Kovrlija[1], Ksenia Menshikh[2], Olivier Marsan[3], Christian Rey[3], Christèle Combes[3], Janis Locs[1,4], Dagnija Loca[1,4*]

[1]Rudolfs Cimdins Riga Biomaterials Innovation and Development Centre, Institute of General Chemical Engineering, Faculty of Materials Science and Applied Chemistry, Riga Technical University, Pulka 3, LV-1007 Riga, Latvia

[2]Center for Translational Research on Autoimmune and Allergic Disease—CAAD, Department of Health Sciences, Università del Piemonte Orientale, 28100 Novara, Italy

[3] CIRIMAT, Université de Toulouse, CNRS, Toulouse INP - ENSIACET, 4 allée Emile Monso, CEDEX 4, 31030 Toulouse cedex 4, France

[4]Baltic Biomaterials Centre of Excellence, Headquarters at Riga Technical University, LV-1007 Riga, Latvia

***corresponding author:** Fax: +37167089619, Tel.: +371 67089628, e-mail: dagnija.loca@rtu.lv

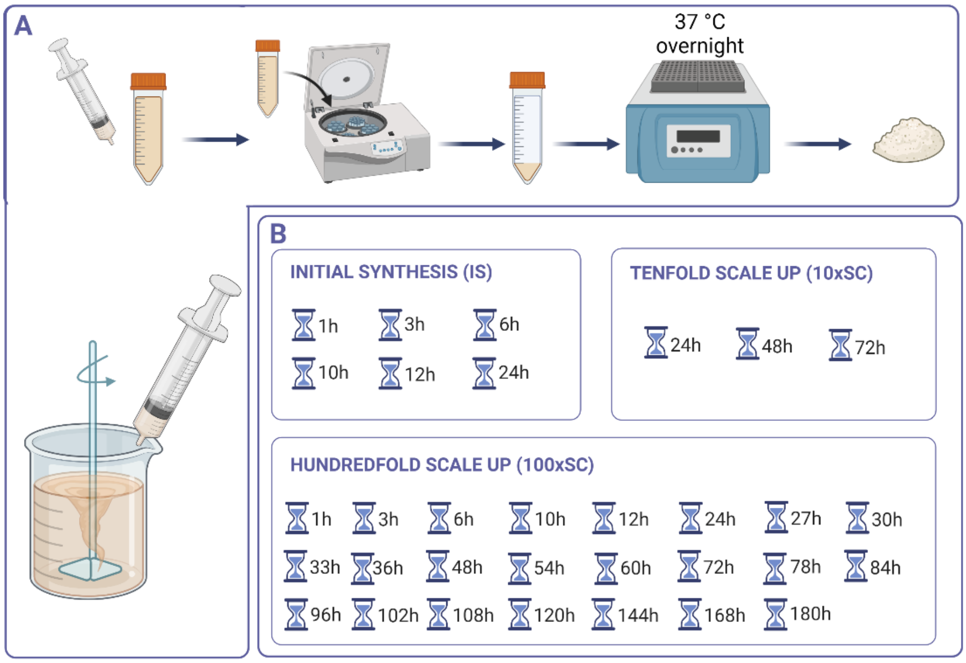

Figure S1. OCP formation from α-TCP suspension – follow-up methodology: A) Acquisition of the samples needed for determining the OCP formation B) Observed time points in the formation of OCP from α-TCP depending on the scale-up level (Created in BioRender.com).

Table S1. List of all the analyzed samples collected at different time points and their presentation within the main manuscript and the supplementary information (S).

<table>
<tr><th colspan="3">Main manuscript</th><th colspan="3">Supplementary information (S)</th></tr>
<tr><th>Initial synthesis (IS)</th><th>Tenfold scale-up (10xSC)</th><th>Hundredfold scale-up (100xSC)</th><th>Initial synthesis (IS)</th><th>Tenfold scale-up (10xSC)</th><th>Hundredfold scale-up (100xSC)</th></tr>
<tr><td rowspan="16">24 h
final OCP</td><td rowspan="16">72 h
final OCP</td><td rowspan="2">1 h</td><td rowspan="3">1 h</td><td rowspan="6">24 h</td><td>3 h</td></tr>
<tr><td>6 h</td></tr>
<tr><td rowspan="2">24 h</td><td>10 h</td></tr>
<tr><td rowspan="3">3 h</td><td>12 h</td></tr>
<tr><td rowspan="2">30 h</td><td>27 h</td></tr>
<tr><td>33 h</td></tr>
<tr><td rowspan="2">48 h</td><td rowspan="3">6 h</td><td rowspan="5">48 h</td><td>36 h</td></tr>
<tr><td>54 h</td></tr>
<tr><td rowspan="2">78 h</td><td>60 h</td></tr>
<tr><td rowspan="3">10 h</td><td>72 h</td></tr>
<tr><td rowspan="2">96 h</td><td>84 h</td></tr>
<tr><td rowspan="5">72 h
final OCP</td><td>102 h</td></tr>
<tr><td rowspan="2">144 h</td><td rowspan="4">12 h</td><td>108 h</td></tr>
<tr><td>120 h</td></tr>
<tr><td rowspan="2">180 h
final OCP</td><td rowspan="2">168 h</td></tr>
<tr></tr>
</table>

1. α-TCP powder phase and composition characterization

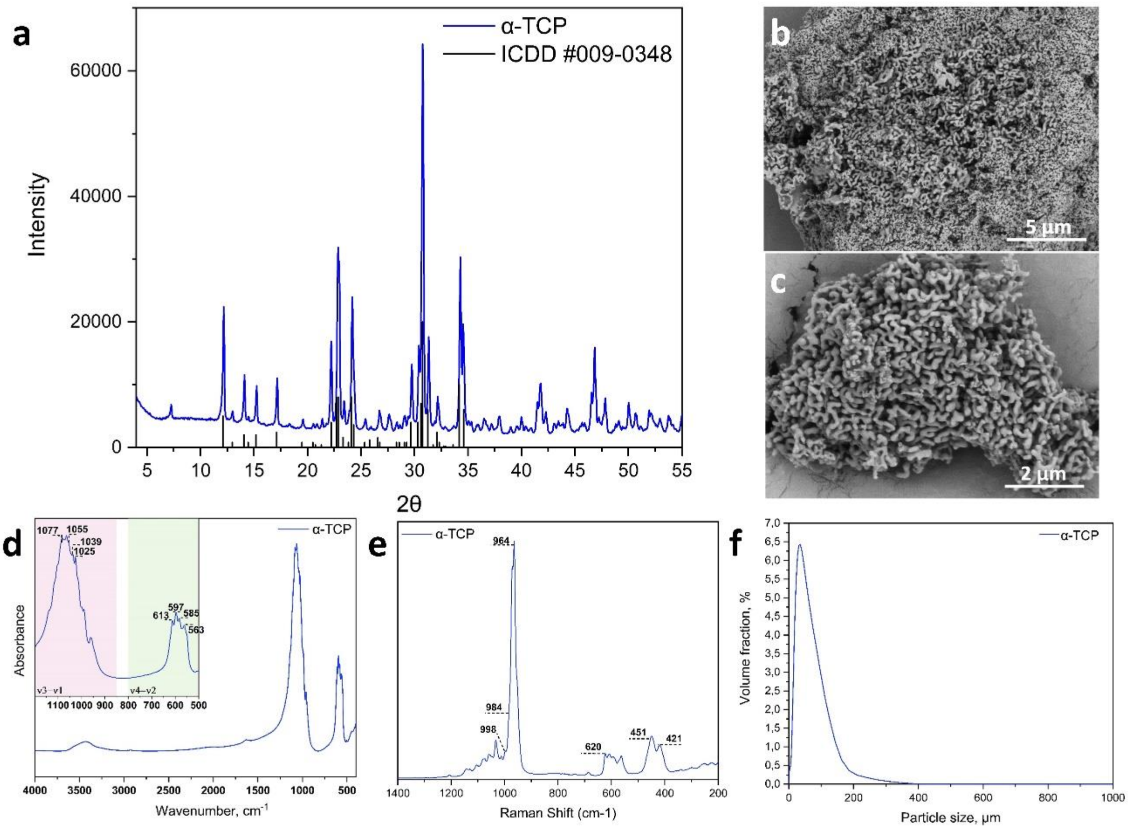

Figure S2. Characterization of the starting α-TCP powder by (a) XRD, (b and c) SEM, (d) FTIR and (e) Raman spectroscopy, and (f) laser granulometry. The corresponding #009-0348 in (a) simulated α-TCP

pattern from the ICDD entry. Pink shading in d) corresponds to $_{1,3}$ PO_4 region and green shading in d) corresponds to $\nu_{2,4}$ PO_4 region.

2. X-RAY DIFFRACTION ANALYSIS

2.1 XRD Initial synthesis (IS)

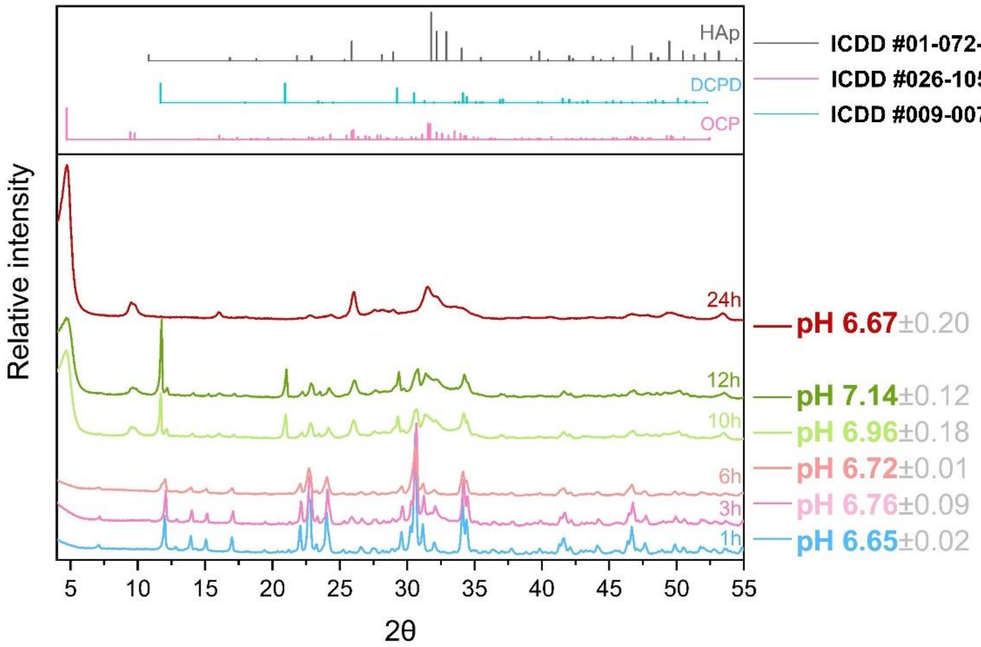

Figure S3. XRD pattern of the specimens collected at different time points during 24 h of IS, with principal phase reference XRD patterns attained from ICDD (#009-0348 for α-TCP; #026-1056 for OCP, #01-072-1243 for HAp, #009-0077 for DCPD).

2.2 XRD Tenfold scale-up (10xSC)

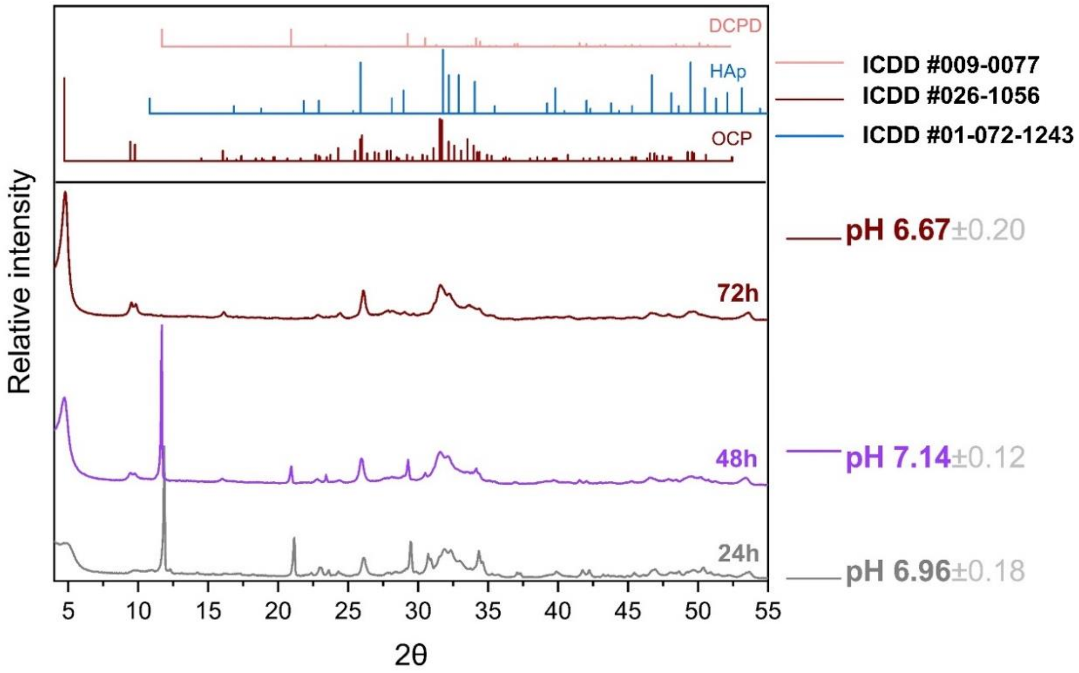

Figure S4. XRD pattern of the specimens collected at different time points during 72 h of 10xSC, with principal phase reference XRD patterns attained from ICDD (#009-0348 for α-TCP; #026-1056 for OCP, #01-072-1243 for HAp, #009-0077 for DCPD).

2.3 XRD Hundredfold scale-up (100xSC)

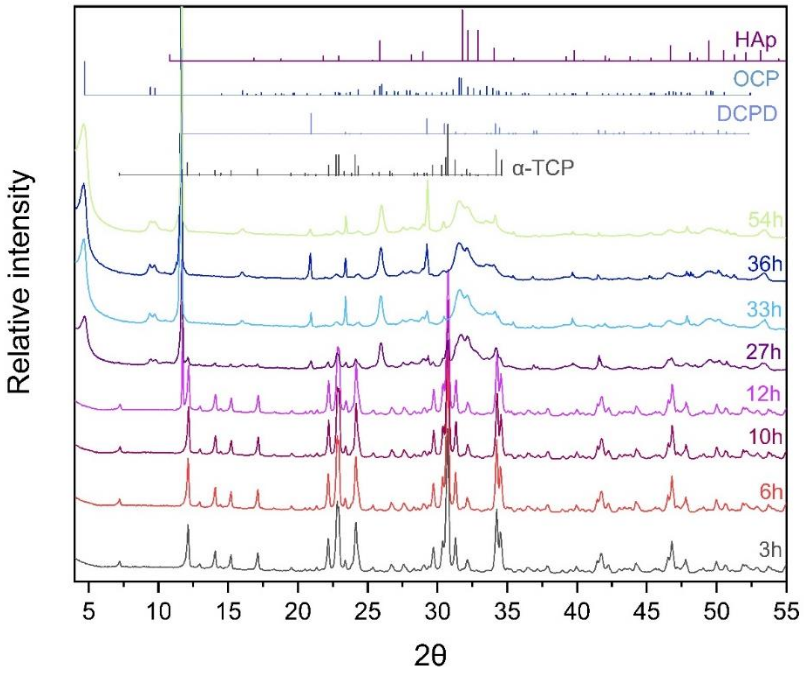

Figure S5. XRD pattern of the specimens collected at different time points from 3 to 54 h during 100xSC and principal phase reference XRD patterns attained from ICDD (#009-0348 for α-TCP; #026-1056 for OCP, #01-072-1243 for HAp, #009-0077 for DCPD).

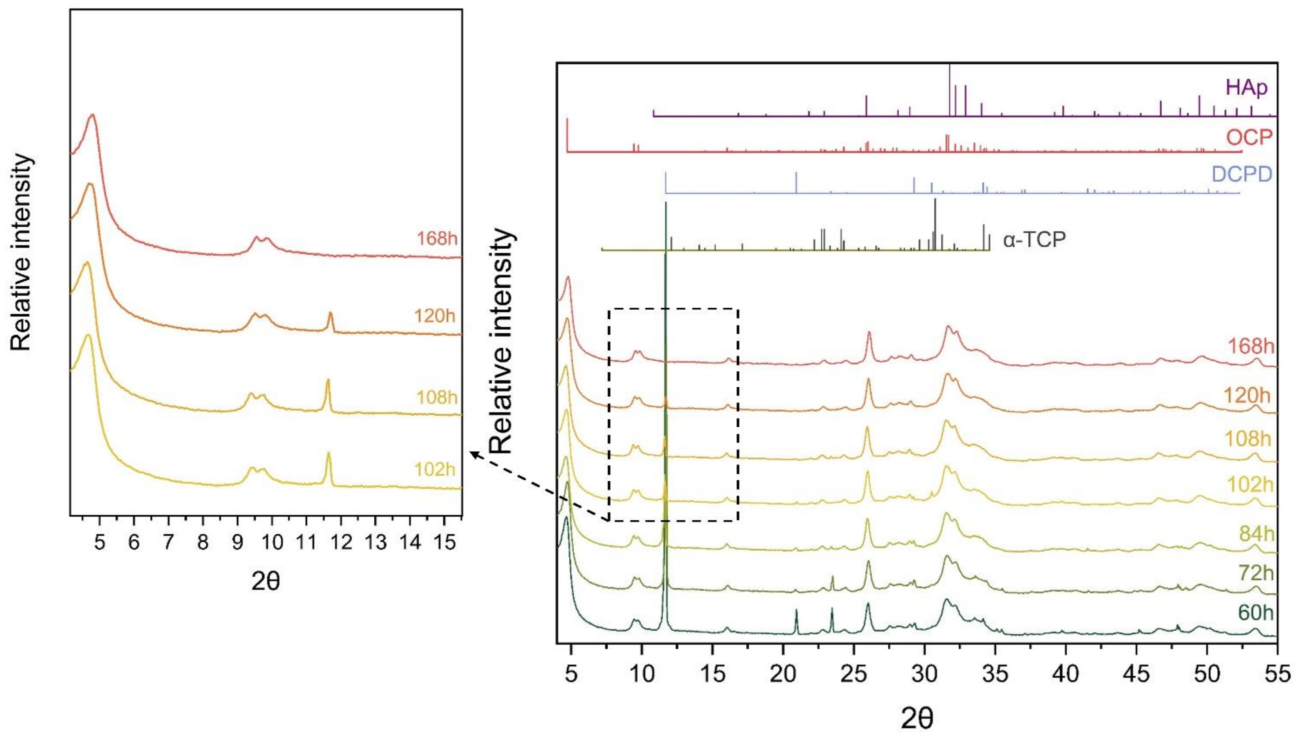

Figure S6. XRD pattern of the specimens collected from 60 to 168 h during 100xSC, and principal phase reference XRD patterns attained from ICDD (#009-0348 for α-TCP; #026-1056 for OCP, #01-072-1243 for HAp, #009-0077 for DCPD).

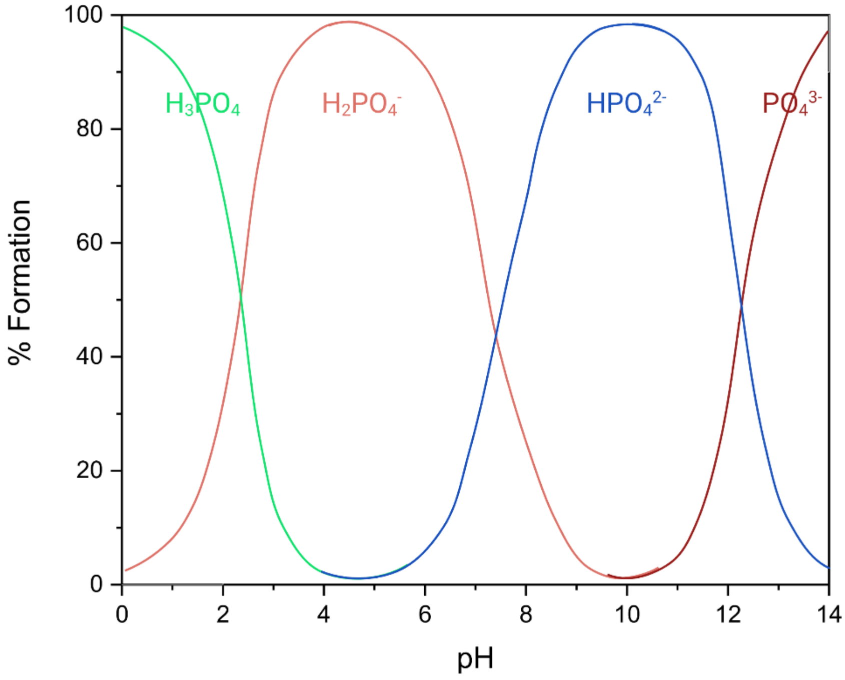

Figure S7. Speciation of orthophosphate species according to the pH. Drawing adopted from Wikipedia.

3. FTIR SPECTROSCOPY ANALYSIS

Table S2. FTIR and Raman band assignments for OCP, α-TCP and DCPD (s-strong, m-medium, sh-shoulder, b-broad, w-weak, v-very, *also ν_3PO_4 stretch) [34,41–45].

Band assignment	α-TCP		DCPD		OCP	
	FTIR	Raman	FTIR	Raman	FTIR	Raman
ν_2 HPO_4(5) bending						356 w<br>413 m, sh
ν_2 PO_4 bend	415 w<br>454 w	421 w<br>451 w	400 m<br>418 sh	381 w<br>411 w	449 vw<br>466 vw	426 m<br>450 m
ν_4 HPO_4 bend					524 w	527 w<br>556 vw<br>578 m
ν_4 PO_4 bend	551 m<br>563 m<br>585 m<br>597 m	577<br>593<br>610 w<br>620 w	526 s<br>577 m	525 w<br>588 w	560 m<br>578 m<br>601 m	578 m<br>591 m<br>609 mw<br>618 sh
HPO_4(5) [P-(OH) stretch]					872 w	879 w
HPO_4(6) [P-(OH) stretch]			872 m	878 m	917 w	916 w
ν_1 PO_4 stretch	954 m	964 s<br>976 s	987 s	986 s	962 w<br>967 w	957 vs<br>966 s
ν_1 HPO_4 stretch					1000vw	1005 w<br>1010sh
ν_3 PO_4 stretch	984 s<br>997 s<br>1013 s<br>1025 s<br>1039 s<br>1055 s	1015 w<br>1027 vw<br>1058 vw<br>1077 vw	1000sh<br>1060 s<br>1070 s	1061 m<br>1079 w	1023 s<br>1037 s<br>1055 s<br>1077 s<br>1093 s	1011 m<br>1026vw<br>1036vw<br>1048 w<br>1079vw
ν_3 HPO_4					1077 s*<br>1093 s*	1079vw
ν_3 HPO_4(5) stretch					1108 s	1109vw
ν_3 PO_4 stretch				1119vw		
ν_3 HPO_4(6) stretch					1121 s*	
ν_3 PO_4 stretch			1132 s			
ν_3 HPO_4 stretch					1138vw	
HPO_4(5) (OH in-plane bend)					1193 w	
ν_3 PO_4 stretch			1215 m			
HPO_4(6) (OH in-plane bend)					1295vw	
HPO_4(6) (OH stretch)					2400 vw,b	
ν_s, ν_{as} stretch of H-bonded H_2O			3145 s<br>3278 s<br>3451 s<br>3500vb		3500vb	

3.1 Initial synthesis (IS)

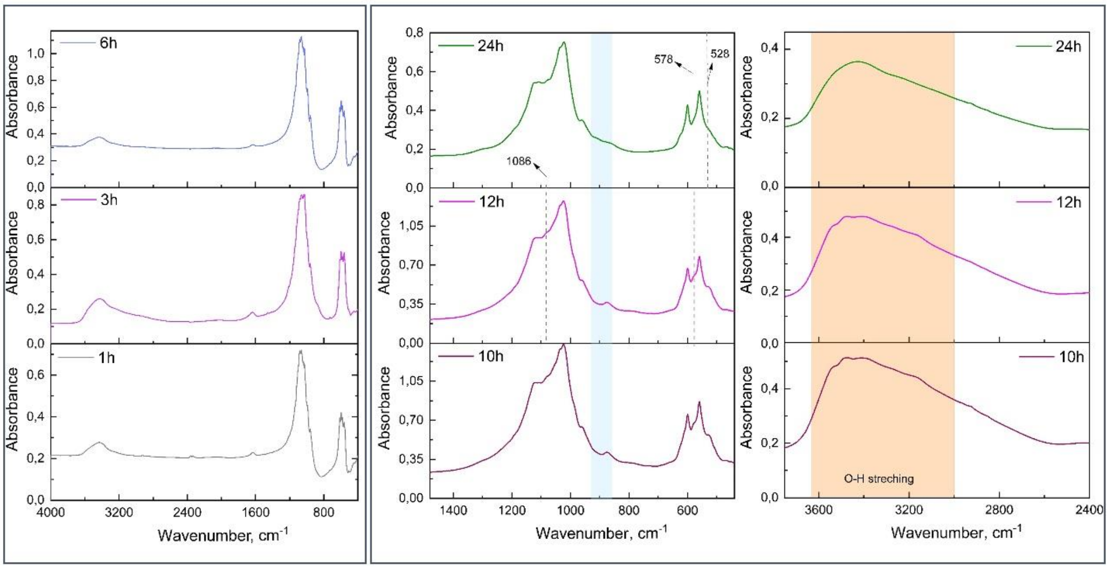

Figure S8. FTIR absorbance spectrum of specimens collected at different time points during the 24 h of IS. Light blue shadowed area is marking the narrow range of HPO_4 (5, 6) P-OH stretch and light orange shadowed area of O-H stretching.

3.2 Tenfold scale up (10xSC)

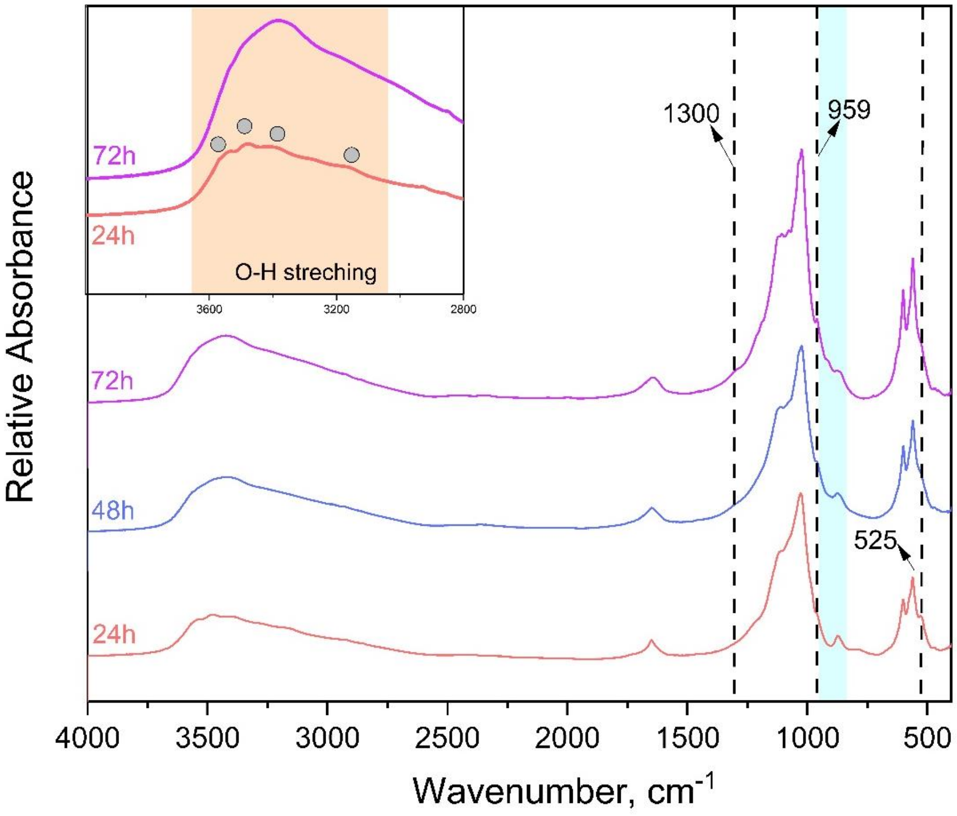

Figure S9. FTIR absorbance spectrum of powders collected at 24, 48 and 72 h of 10xSC. Blue shadowed area is marking the narrow range of HPO_4 (5, 6) P-OH stretch and light orange shadowed area corresponds to O-H stretching (gray circle DCPD).

3.3 Hundredfold scale up (100xSC)

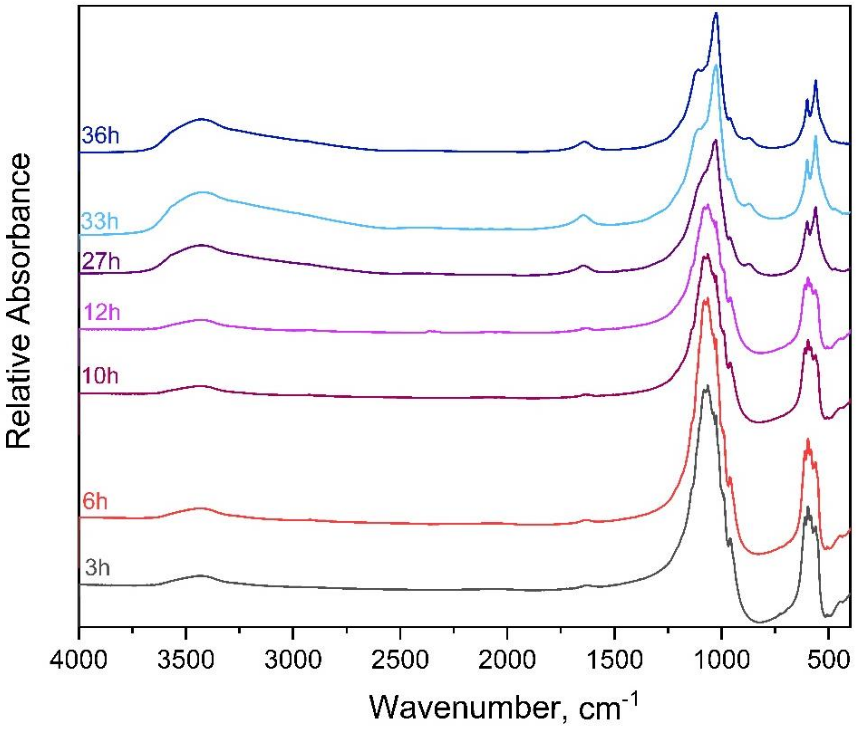

Figure S10. FTIR absorbance spectrum of powders collected at different time points during 36 h of 100xSC. The missing time points (1 h, 24 h) have been disclosed in the main manuscript.

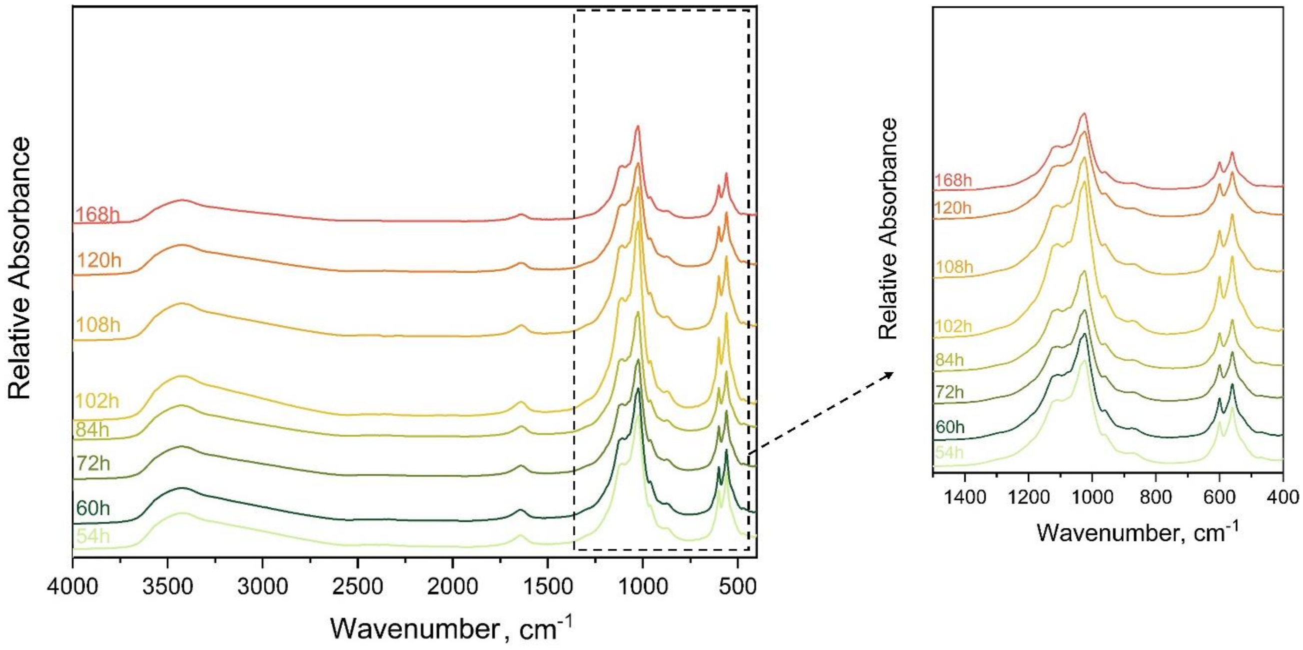

Figure S11. FTIR absorbance spectrum of specimens collected at different time points during the period from 54 h – 168 h of 100xSC. The missing time points (78 h, 96 h, 144 h and 180 h) have been disclosed in the main manuscript.

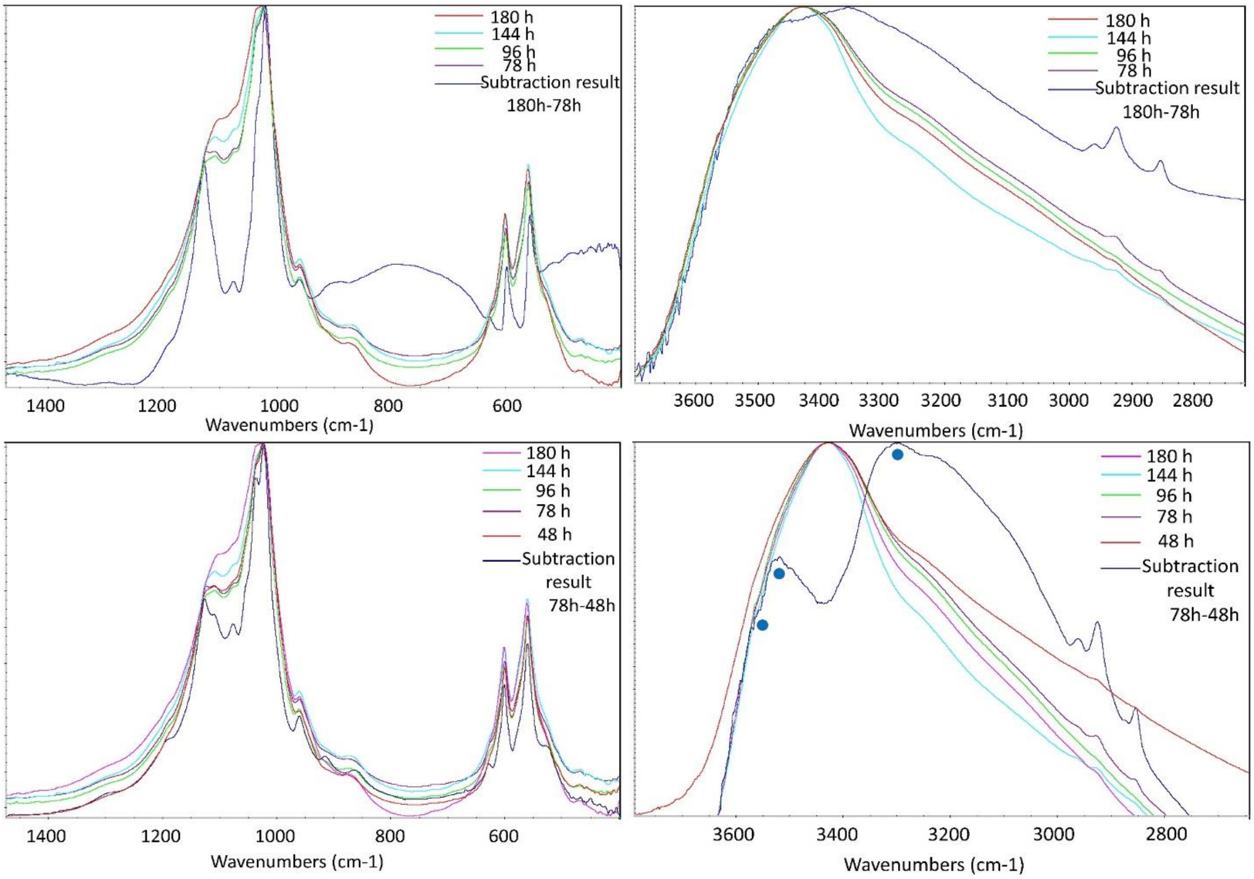

Figure S12. FTIR subtraction absorbance band between 100xSC 78 h and 180 h samples (upper row) and FTIR subtraction absorbance band between 100xSC 48 h and 78 h samples. Blue circles are marking the possible position of DCPD absorption bands.

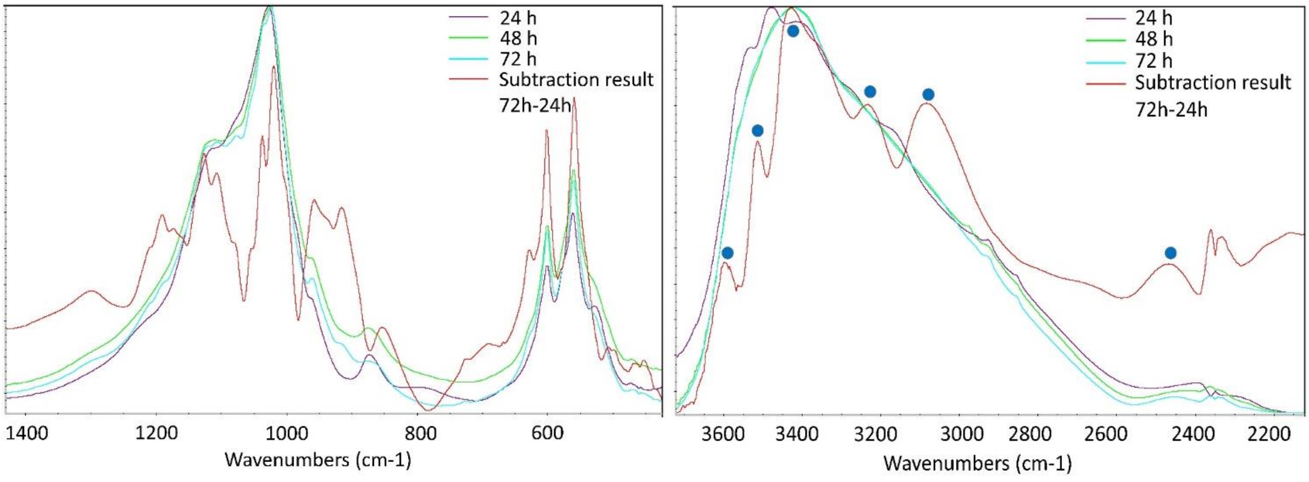

Figure S13. FTIR subtraction absorbance band between 10xSC 24 h and 72 h samples. Blue circles are marking the possible position of DCPD absorption bands.

4. RAMAN SPECTROSCOPY ANALYSIS

4.1 Initial synthesis (IS)

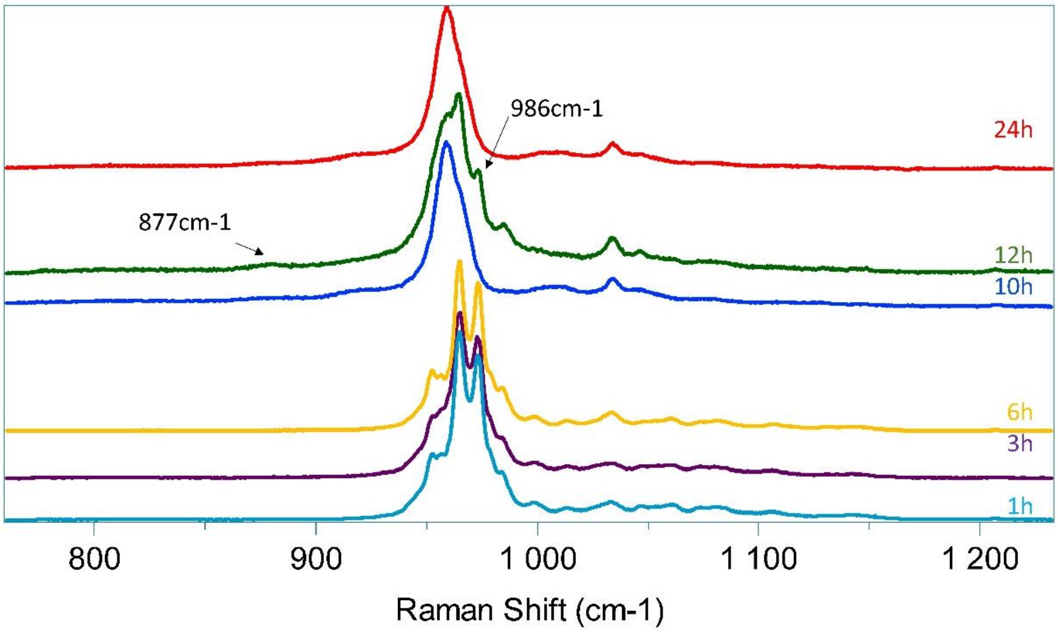

Figure S14. Raman spectra of the powders collected at different time points during initial synthesis (IS). The graph displays a close up of ν_1 stretching mode of the P – O vibration with marked band positions at 877 cm^{-1} and 986 cm^{-1}.

4.2 Tenfold scale-up (10xSC)

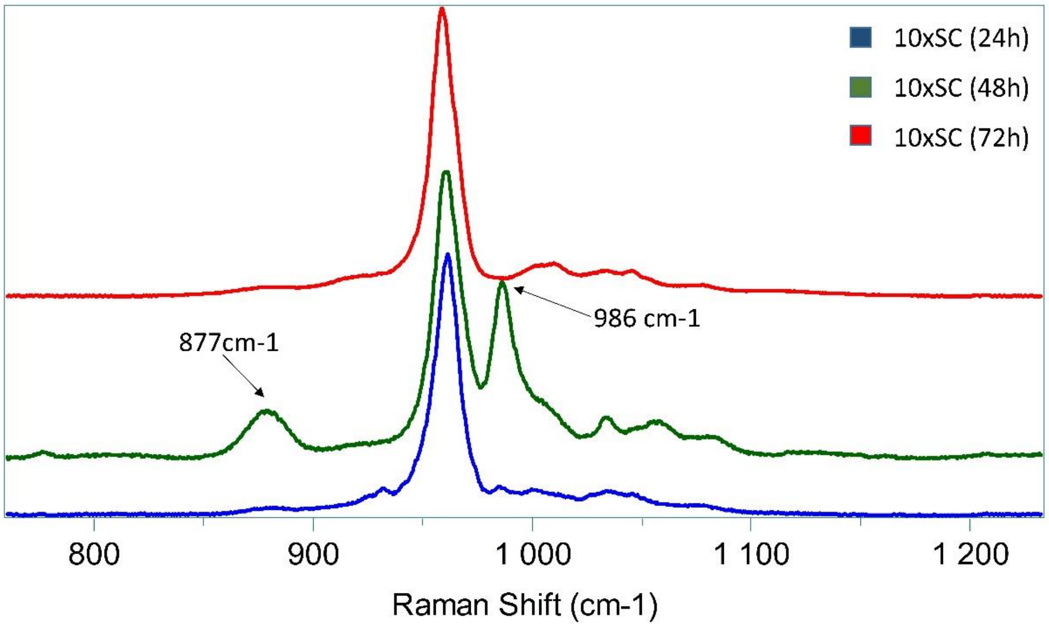

Figure S15. Raman spectra of the powder evolution as a function of time in 10xSC synthesis. The graph displays a close up of ν_1 stretching mode of the P – O vibration with marked band positions at 877 cm^{-1} and 986 cm^{-1}.

4.3 Hundredfold scale-up (100xSC)

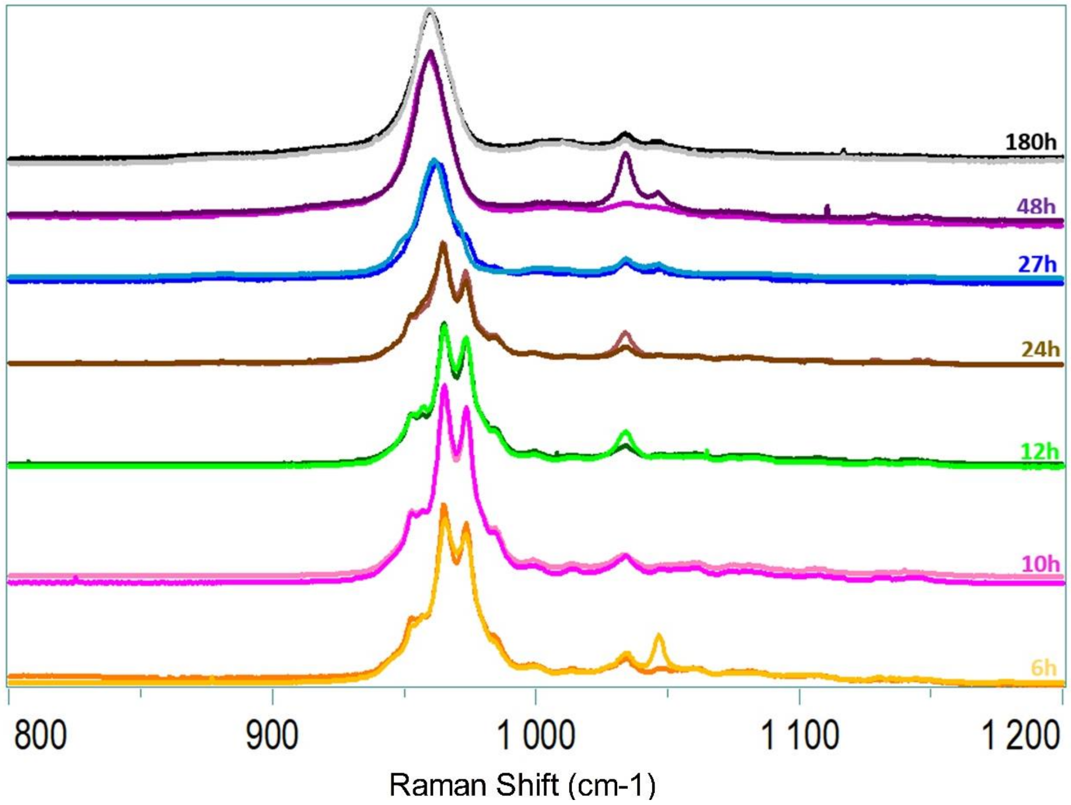

Figure S16. Raman spectra comparison between micro (light color) and macro spot (dark color) of the powders collected during 100xSC.

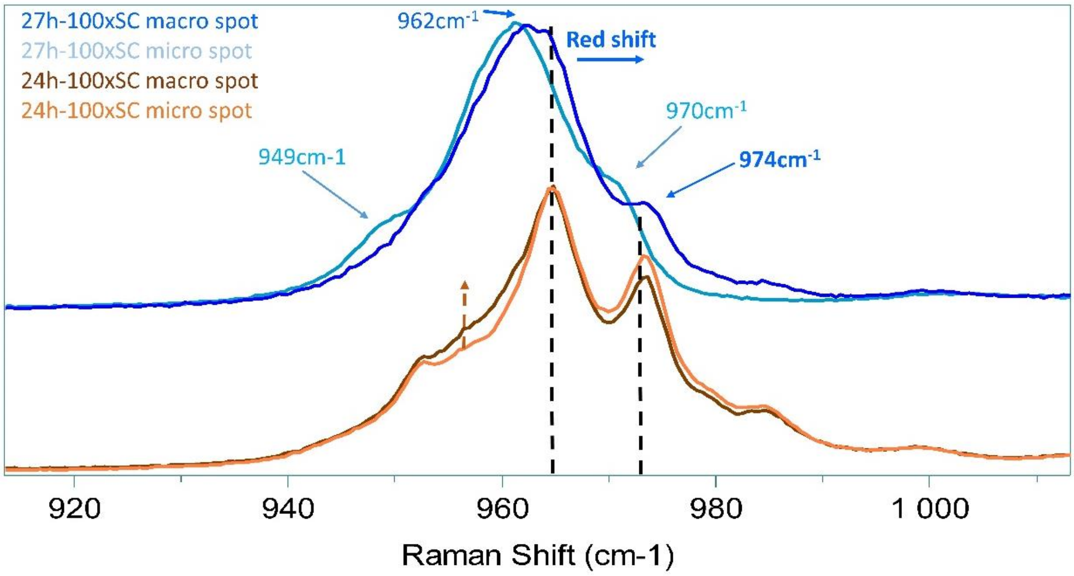

Figure S17. Raman spectra comparison between micro (light color) and macro spot (dark color) of the powders collected after 24 h and 27 h during 100xSC.

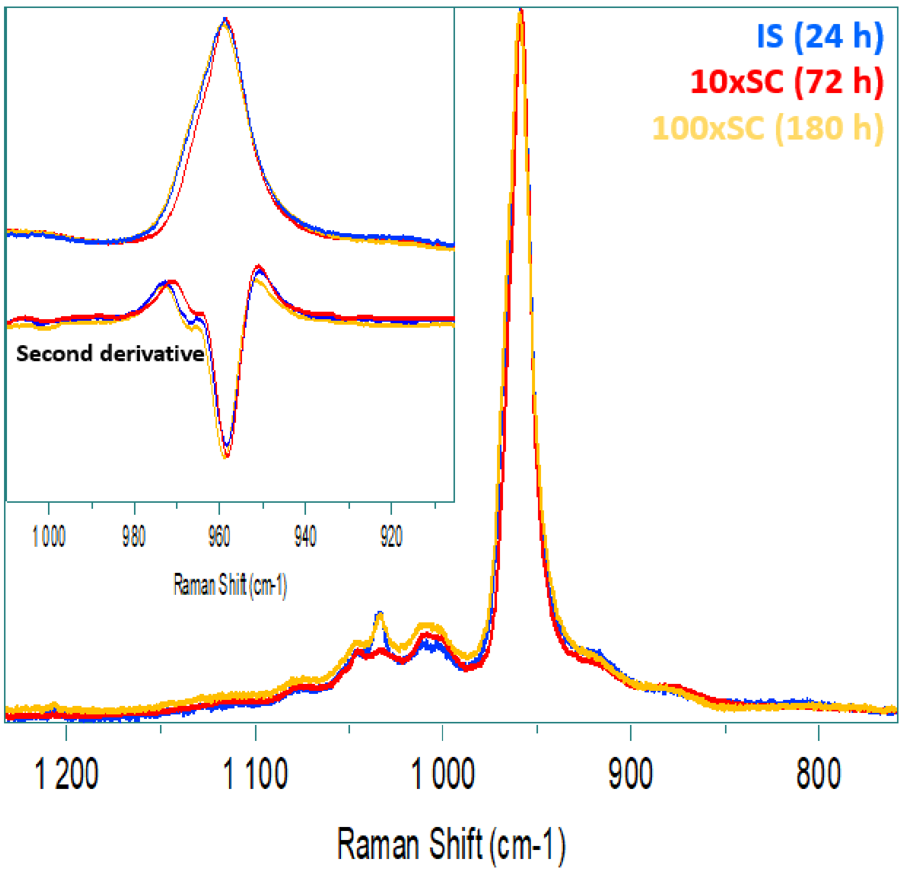

Figure S18. Raman spectra of final OCP phases obtained in three levels of scale-up and the representation of the second derivative of the most prominent band at 958 cm^{-1}.

5. SCANNING ELECTRON MICROSCOPY ANALYSIS

5.1 Initial synthesis (IS)

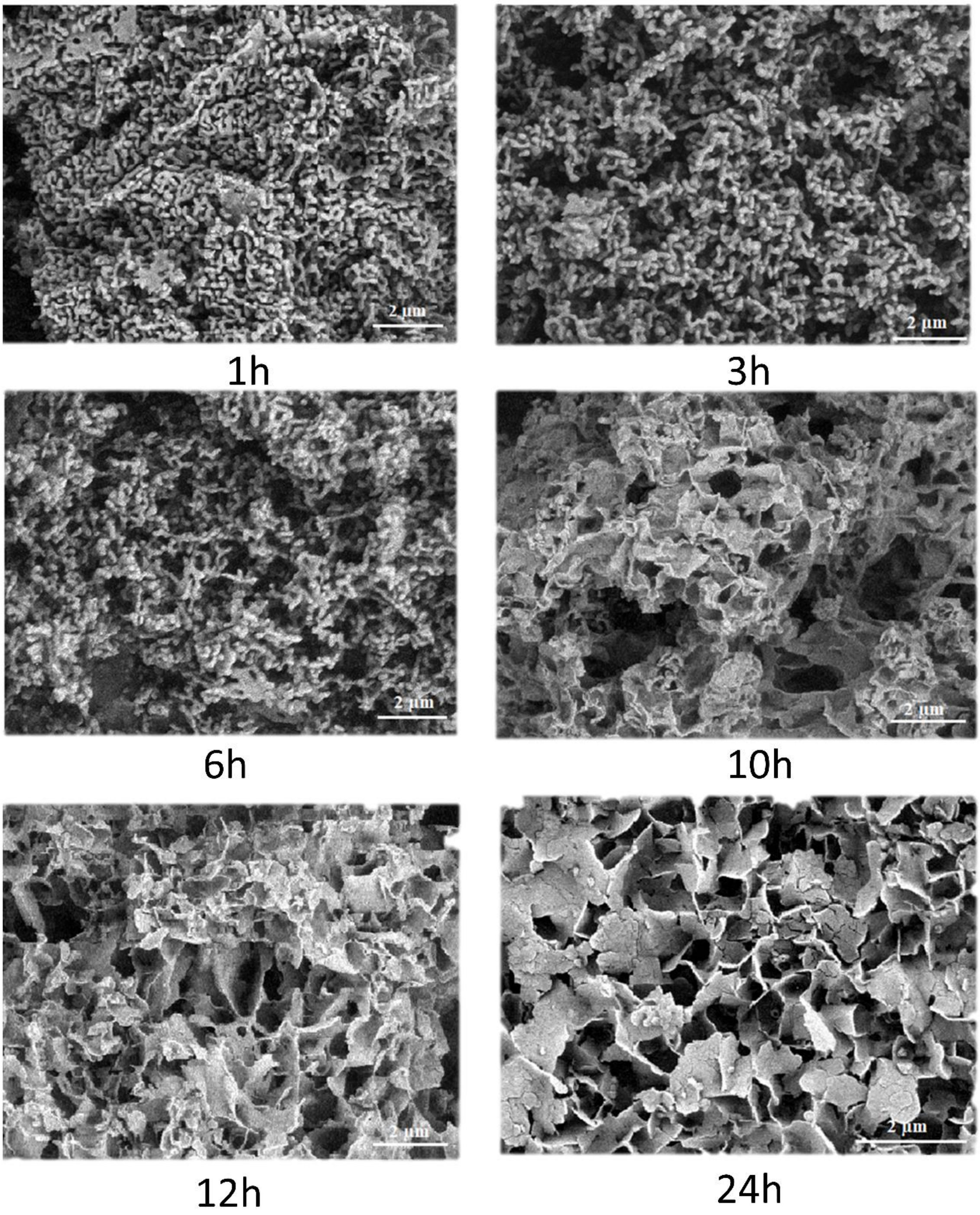

Figure S19. Scanning electron micrographs of α-TCP transformation to OCP (initial synthesis), observed at specific time points indicated below each SEM image. Scale bar 2 μm.

5.2 Tenfold scale-up (10xSC)

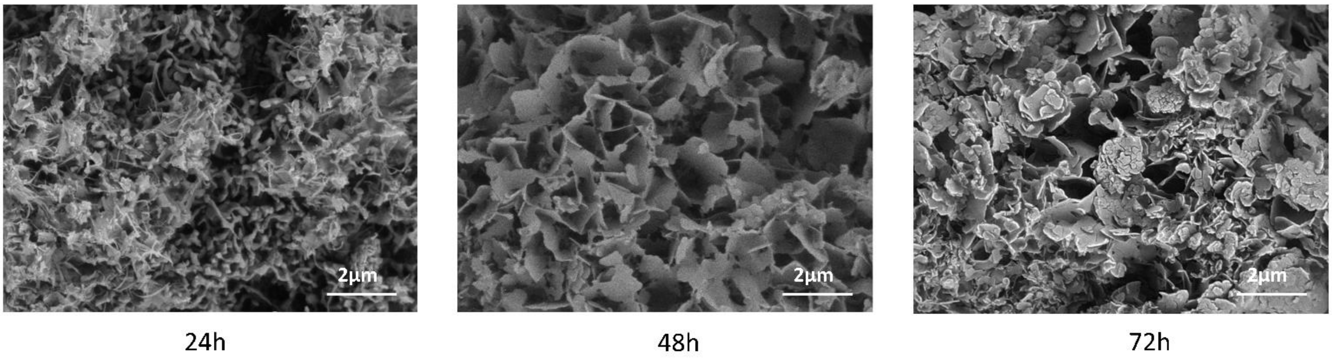

Figure S20. Scanning electron micrographs of α-TCP transformation to OCP (10xSC), observed at specific time points indicated below each SEM image. Scale bar 2 μm.

6. LASER GRANULOMETRY

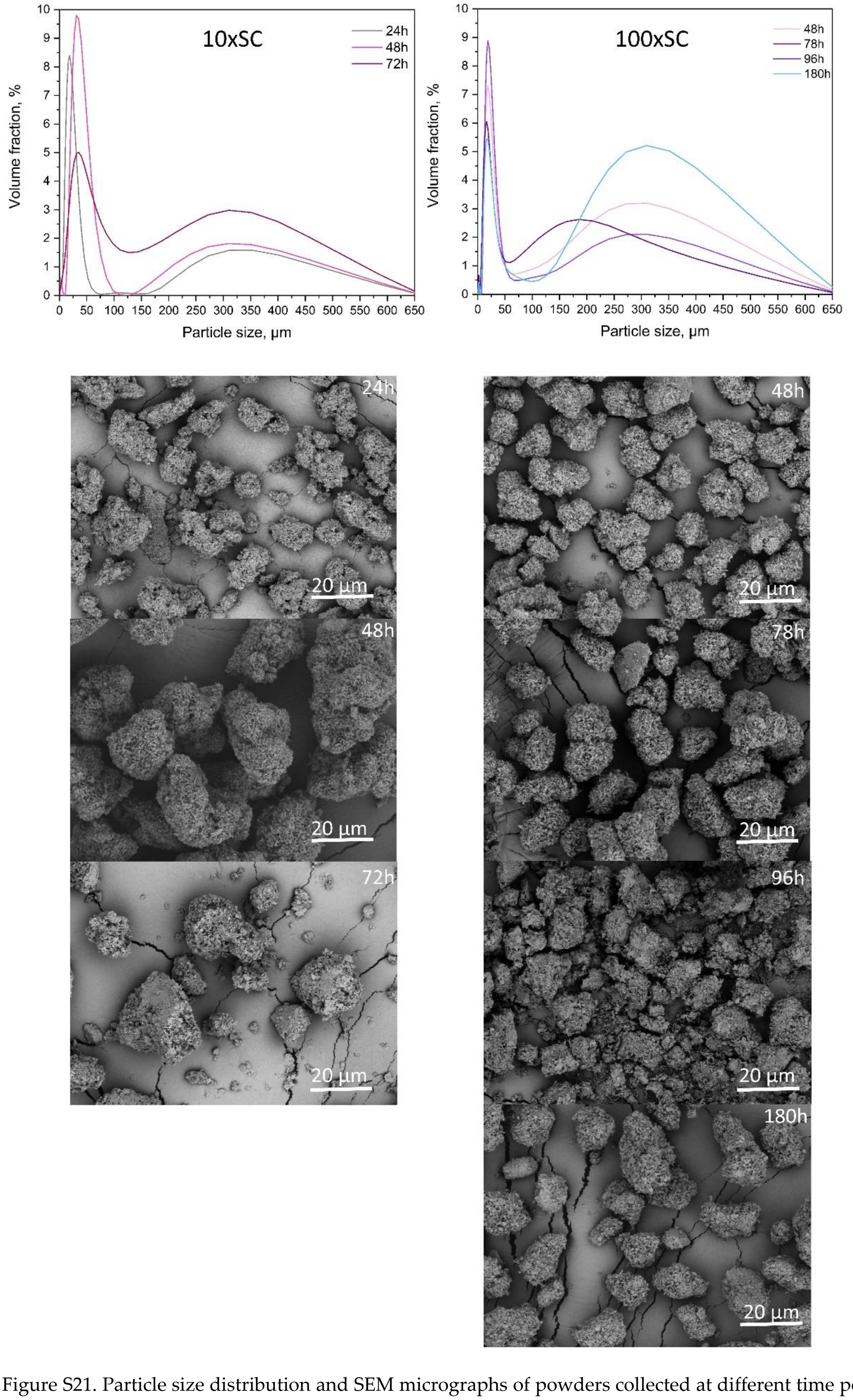

Figure S21. Particle size distribution and SEM micrographs of powders collected at different time points during 10xSC and 100xSC, showing the presence of agglomerated crystals (scale bar 20 µm).

7. CELL PHENOTYPE MORPHOLOGY

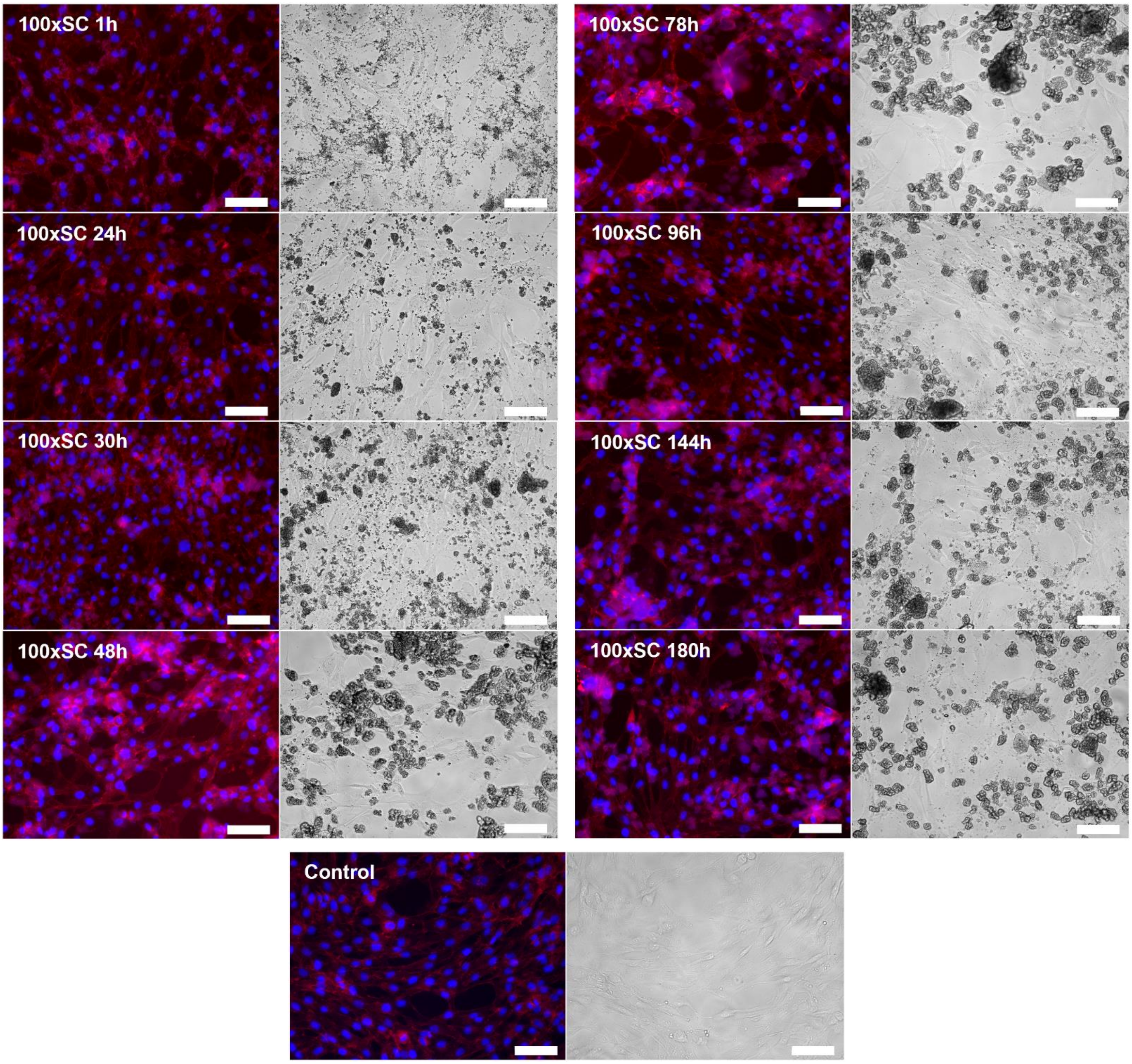

Figure S22. hBMSCs morphology on the third day of cultivation in direct contact with hundredfold synthesis OCP samples in the concentration of 0.5 mg/mL. *Control* represents cells on polystyrene. Immunofluorescent (DAPI – blue, phalloidin – red) and bright-field microscopy. Image bar scale: 125 μm.

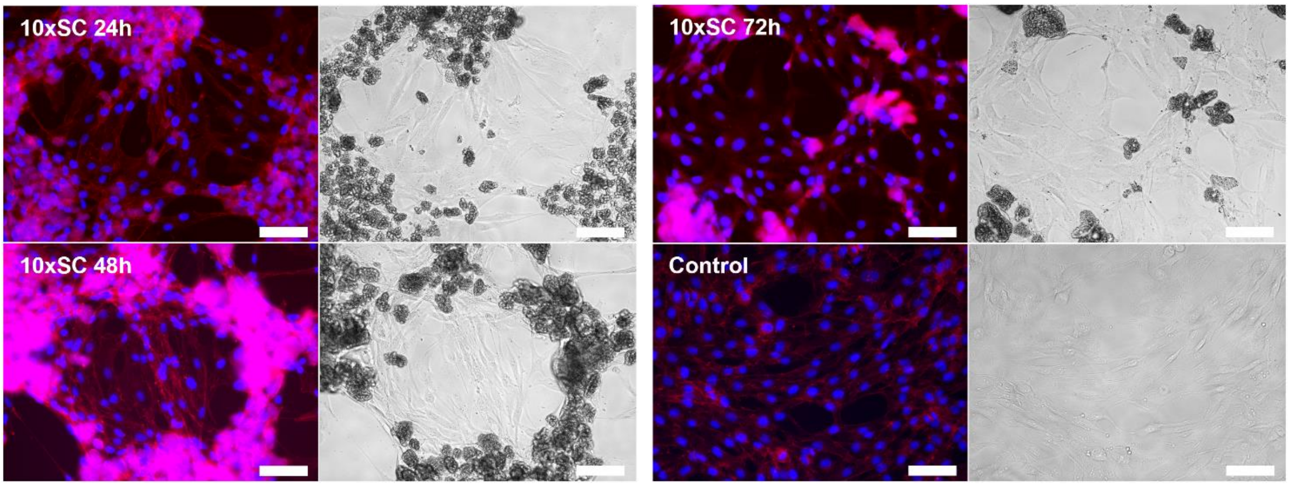

Figure S23. hBMSCs morphology on the third day of cultivation in direct contact with ten-fold synthesis OCP samples in the concentration of 0.5 mg/mL. *Control* represents cells on polystyrene. Immunofluorescent (DAPI – blue, phalloidin – red) and bright-field microscopy. Image bar scale: 125 μm.

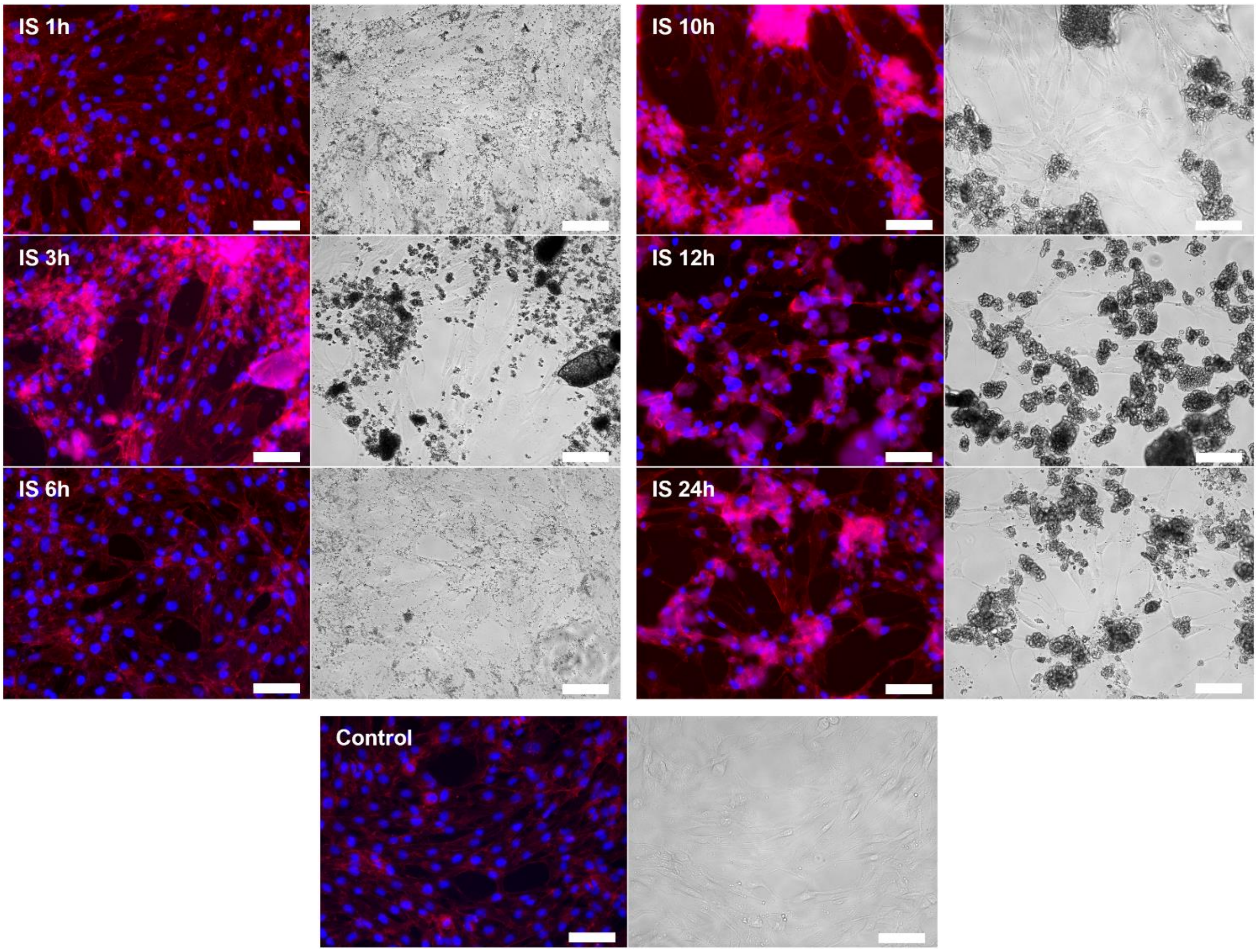

Figure S24. hBMSCs morphology on the third day of cultivation in direct contact with initial synthesis OCP samples in the concentration of 0.5 mg/mL. *Control* represents cells on polystyrene. Immunofluorescent (DAPI – blue, phalloidin – red) and bright-field microscopy. Image bar scale: 125 μm.
